# Tetrazine Functionalized Graphene Enables Capture of Ultra‐Low Concentrations of Biomacromolecules

**DOI:** 10.1002/smll.202513723

**Published:** 2026-03-25

**Authors:** Ravindra K. Gupta, Hanbin Jeong, April Goehring, Eric Gouaux, Maggie He

**Affiliations:** ^1^ Department of Chemistry and Biochemistry University of Arkansas Fayetteville Arkansas USA; ^2^ Vollum Institute Oregon Health and Science University Portland Oregon USA; ^3^ Howard Hughes Medical Institute Oregon Health and Science University Portland Oregon USA

**Keywords:** bioorthogonal, capture, diazonium chemistry, graphene, nanobody, surface, tetrazine ligation

## Abstract

Monolayer graphene has great potential for a range of technological and biomedical applications. To achieve this potential, however, selective functionalization of the surface using mild chemical methods that yield spatially and chemically well‐defined functional groups is needed. Here, we describe a method to densely and uniformly modify the surface of graphene using a diazonium salt containing a tetrazine moiety, a functional group amenable to bioorthogonal chemistry. The resulting functionalized graphene efficiently undergoes inverse electron demand Diels‐Alder reactions with *trans*‐cyclooctene (TCO) coupled compounds, including a small organic molecule and an anti‐GFP nanobody. The nanobody decorated graphene readily captures EGFP tagged apoferritin (EGFP apo‐Ftn) and by following the binding by total internal fluorescence microscopy, we demonstrate that the functionalized graphene can robustly capture EGFP apo‐Ftn molecules at a concentration of 2 pm.

## Graphical abstract

A novel method for covalent modification of monolayer graphene utilizing tetrazine containing diazonium salt is demonstrated. The modified graphene bearing tetrazine moieties undergoes inverse electron demand Diels‐Alder reactions with *trans*‐cyclooctene‐linked molecules, enabling bioorthogonal attachment of nanobodies that selectively capture EGFP‐tagged proteins at concentrations as low as 2 pm, highlighting its potential for biosensing and nanotechnology applications.

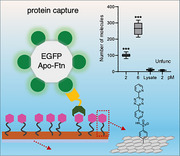

## 1 Introduction

Monolayer graphene is a remarkable material due to its high electrical and thermal conductivity, mechanical strength, and transparency to both visible light and electrons, and thus is utilized in a wide array of technologies, from biosensors to filtration membranes [1]. To most effectively harness the favorable characteristics of graphene, however, methods to chemically modify the surface with an array of discrete functional groups and chemistries are needed. While approaches to noncovalently functionalize the graphene surface with various planar (aromatic derivatives, most commonly typified by 1‐pyrene compounds [2, 3, 4, 5, 6]) have shown utility in several applications, such methods do not offer the same degree of stability and spatial control as covalent functionalization. There are methods to modify the graphene surface through oxidation to produce graphene oxide, which enables covalent functionalization. However, existing graphene oxide preparation techniques typically involve harsh chemical conditions, complex purification steps, and result in uncontrolled defect formation [7].

Aryl diazonium salts offer an attractive vehicle for the functionalization of monolayer graphene [8], not only because the electron transfer reaction can proceed under mild conditions, but also because the chemistry is compatible with multiple functional groups and the resulting modification of the graphene surface is extensive and uniform [8]. The initial reactions by Pinson's group, showing the derivatization of carbon surfaces by (4‐nitrophenyl)diazonium tetrafluoroborate yielding a nearly close‐packed monolayer of 4‐nitrophenyl groups [8], to more recent studies utilizing various diazonium salts, have demonstrated the utility of diazonium‐mediated modification of carbon and graphene surfaces [9]. Here, we have chosen to utilize diazonium chemistry, in combination with compounds harboring a highly efficient bioorthogonal reaction partner, to functionalize the surface of monolayer graphene.

Surfaces designed for biomedical applications, such as diagnostics, biosensing, and molecular imaging, are particularly valuable when they can be readily and efficiently tailored with diverse chemical functionalities or biomolecules [10, 11]. In many biological systems, key protein targets, including biomarkers, signaling molecules, and therapeutic agents, are often present at very low concentrations [12]. This makes it essential for surface chemistries to operate with both high efficiency and selectivity under dilute conditions. A functionalized surface capable of reacting with and capturing molecules at low abundance not only minimizes nonspecific binding, but also reduces the consumption of reagents and scarce biomacromolecules. These features are also critical for analyzing complex biological samples while preserving the native structure and activity of fragile proteins [13]. Moreover, achieving efficient reactivity at low concentrations is a key requirement for translating surface modification strategies into scalable, practical, and cost‐effective technologies [10, 14]. Furthermore, surface modifications can include functional groups that, in turn, enable further modification under mild conditions.

Tetrazine has been widely applied in materials, including tetrazine‐tagged linkers enabling post‐synthetic modification of metal–organic frameworks [15, 16], metal–organic cages comprised of dipyridyl tetrazine capable of structural transformations through reactions [17], tetrazine‐linked covalent organic frameworks with photocatalytic and sensing properties [18], tetrazine‐based covalent and porous organic cages for gas separation and molecular recognition [19], and tetrazine‐containing polymers and dynamic polymer networks enabling efficient post‐polymerization click functionalization [20, 21]. Here, we are integrating tetrazine groups onto graphene to enable its application in biomolecule capture.

Tetrazine can be employed in various click reactions [22, 23, 24]. Among these, the inverse electron demand Diels–Alder (IEDDA) reaction between 1,2,4,5‐tetrazine and strained dienophiles is among the fastest known bioorthogonal ligation strategies [24]. Its exceptional rate arises from the combined electronic and structural activation of the two reacting partners. Electron‐withdrawing substituents, such as pyridyl groups on the tetrazine, enhance its reactivity, while ring strain imposed on cyclic dienophiles further accelerates the reaction. Given these considerations, we chose to functionalize the graphene surface with dipyridyl tetrazine groups that have demonstrated both stability and rapid ligation with TCO, a highly strained dienophile [25, 26].

Graphene‐based materials, including chemical vapor deposition (CVD)‐grown graphene and graphene oxide, have been investigated as platforms for molecular capture. Graphene oxide, typically produced through harsh oxidative treatment of graphite or graphene, contains various oxygenated functional groups and structural defects across its basal plane [27, 28, 29]. Functionalization generally involves multiple‐step chemical reactions targeting these oxygenated sites to introduce desired functionalities. While covalent bonding, such as those between the SpyCatcher protein and the SpyTag peptide have been employed [30, 31], the overall surface chemistry of graphene oxide remains poorly defined, with limited control over functional group identity and spatial distribution. In addition, protein particle capture on these surfaces often relies on reversible, noncovalent interactions [32, 33, 34, 35]. Covalent grafting via diazonium salts has been previously explored. However, these conventional approaches typically rely on either incorporating a carboxylic acid group into the diazonium salt or introducing an aniline moiety into the protein. The former strategy requires activation of the carboxylic acid with coupling agents to allow reaction with amine groups on a protein, while the latter involves in situ diazotization using highly reactive nitrites [36, 37, 38]. In addition, amide couplings generally require relatively high concentrations of reagents and proteins, and they are not specific in complex environments such as cells or cell lysates where amines and carboxylic acids are abundant. Here, we report a one‐step method for covalently functionalizing monolayer graphene on glass with a dipyridyl tetrazine diazonium salt. This diazonium salt functionalization proceeds in DMSO at room temperature without additional reagents, affording a highly defined and reactive surface suitable for subsequent derivatization with a broad range of small organic molecules and biomolecules via rapid bioorthogonal tetrazine ligation. The tetrazine‐functionalized surface demonstrates high efficiency in the tetrazine ligation, allowing the covalent conjugation of a TCO tethered nanobody and subsequent capture of an EGFP tagged protein, even at ultra‐low concentrations.

## 2 Results and Discussion

### 2.1 Tetrazine Ligation Kinetics

**FIGURE 1:**
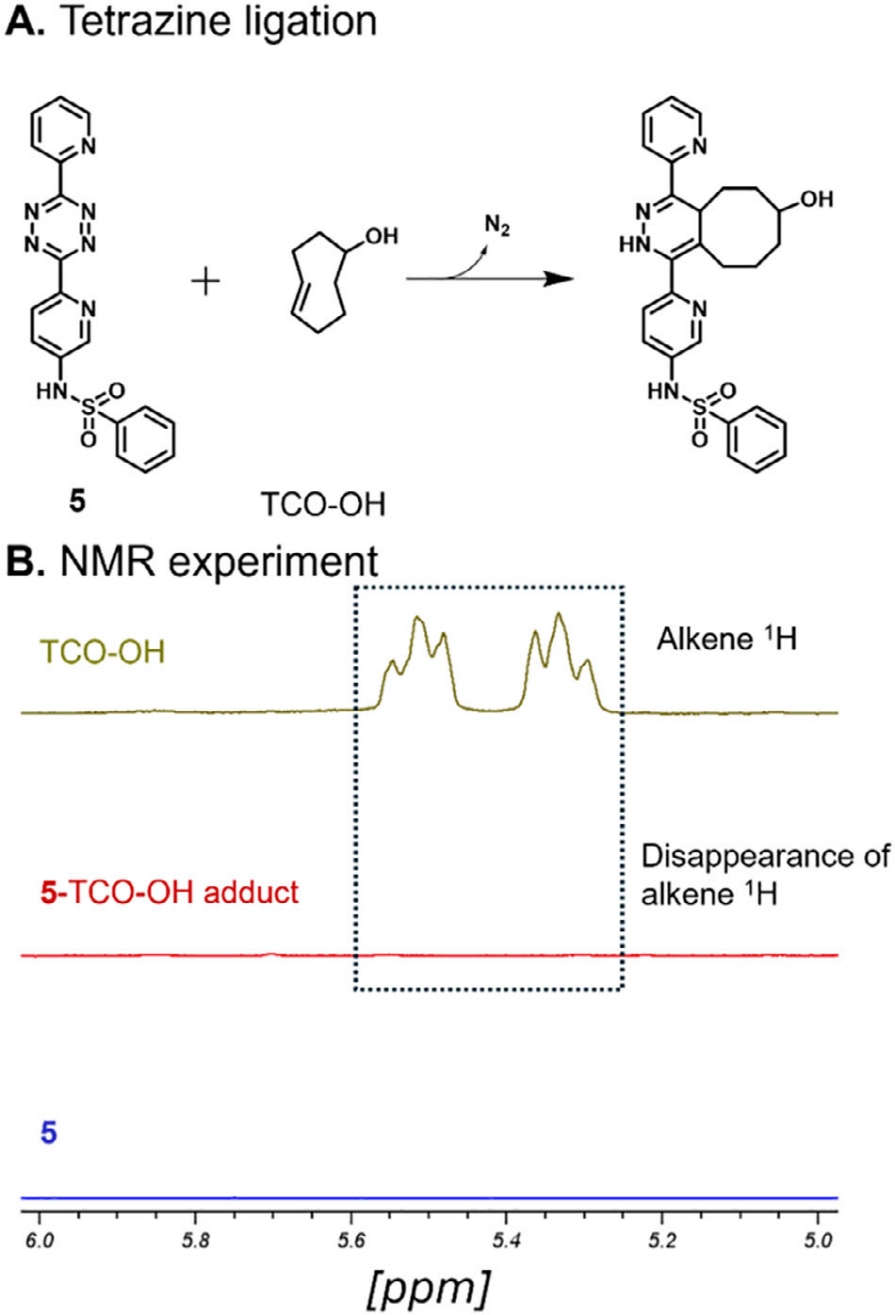
(A) Tetrazine ligation. (B) ^1^H NMR experiment for TCO‐OH and tetrazine **5** reaction after 10 min of mixing both reactants at room temperature.

To verify that the target tetrazine molecule for use in coupling to monolayer graphene will successfully react with a TCO derivative, we synthesized dipyridyl tetrazine **5** and studied its reaction with *trans*‐cyclooctene‐OH (TCO‐OH, Figure 1A) by ^1^H NMR. An equimolar solution of **5** and TCO‐OH was prepared in deuterated dimethyl sulfoxide ((CD_3_)_2_SO). The ^1^H NMR spectrum of TCO‐OH was first recorded and then an equimolar amount of compound **5** was added to the TCO‐OH solution in the NMR tube. The immediate evolution of gas bubbles upon mixing tetrazine **5** with TCO‐OH suggested a rapid reaction between **5** and TCO‐OH. We followed the reaction by ^1^H NMR and after 10 min of mixing the absence of the alkene protons in the spectrum of the TCO‐tetrazine conjugate confirmed that the reaction was complete (Figure 1B; Figure S1 and S2).

To further probe the reactivity of the target tetrazine molecule **5** in reactions at low concentrations, the reaction kinetics with TCO‐OH were conducted under pseudo‐first‐order conditions with the dienophile present at a concentration ten times higher than the diene (Figure S3) [39]. The second‐order rate constant (k_2_) was determined by plotting the pseudo‐first‐order rate constants against the concentration of **5** and performing a linear fit. The calculated k_2_ value was 3228.15 m ^−1^s^−1^, consistent with tetrazine and TCO ligation reactions in a mixed solvent system [26].

### 2.2 Synthesis of the Diazonium Salt

**SCHEME 1:**
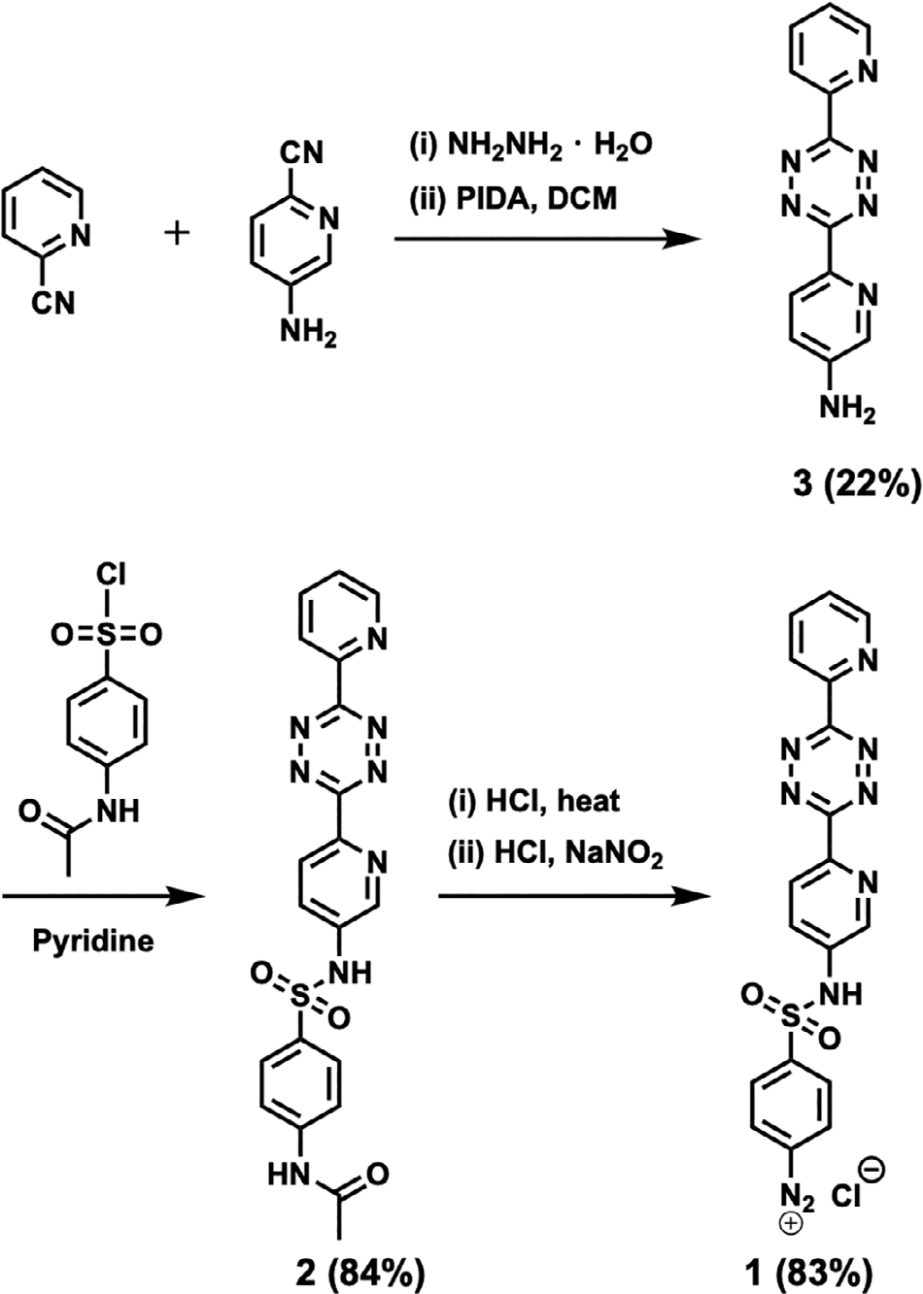
Synthesis of diazonium salt **1**.

With the confirmation that compound **5** has suitable kinetics in the tetrazine ligation reaction, we synthesized tetrazine diazonium salt **1** for the grafting of compound **5** onto graphene. Diazonium salt **1** was synthesized according to previous procedures with modifications (Scheme 1) [40, 41]. Tetrazines, including structurally complex derivatives, can be synthesized through various routes [42]; however, in this work, we chose to employ a traditional synthetic approach. Briefly, condensation of 2‐cyanopyridine and 5‐amino‐2‐pyridinecarbonitrile with hydrazine hydrate followed by oxidation provided dipyridyl tetrazine **3**. Dipyridyl tetrazine **3** was then coupled with 4‐acetamidobenzenesulfonyl chloride to furnish *N*‐acetyl benzenesulfonamide **2**. Deacetylation followed by diazotization in one pot provided the desired diazonium salt **1** in good yield. Complete detailed synthesis and compound characterization can be found in the Supporting Information.

### 2.3 Functionalization and Characterization of Graphene on Glass

**FIGURE 2:**
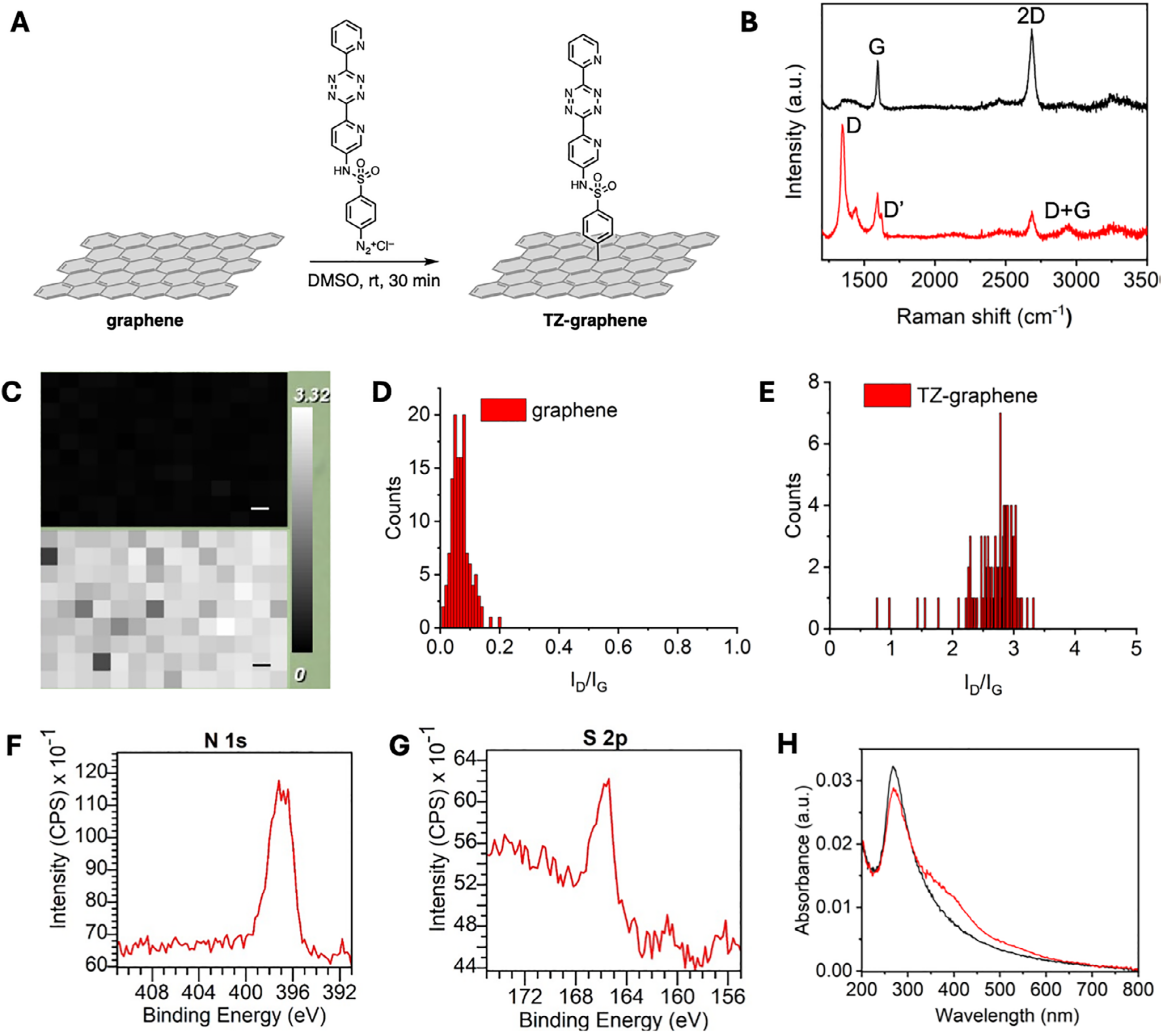
(A) Scheme showing the functionalization of graphene with dipyridyl tetrazine groups; (B) Representative Raman spectra of unfunctionalized graphene (black) and TZ‐graphene (red) on glass; (C) Raman *I_D_/I_G_* ratio map of graphene and TZ‐graphene on glass (Raman map scale bar is 10 µm); (D) Bar graph showing the distribution of *I_D_/I_G_* ratio for graphene on glass; (E) Bar graph showing the distribution of *I_D_/I_G_* ratio for TZ‐graphene on glass. (F,G) XPS of TZ‐graphene showing the N 1s and S 2p regions. (H) UV–vis of graphene (black) and TZ‐graphene (red) on quartz.

Commercially available monolayer graphene produced by CVD was utilized in this study. Monolayers of graphene were transferred to glass slides using poly(methyl methacrylate) (PMMA) (see Methods). After transferring graphene onto glass slides, the graphene on glass was functionalized using diazonium salt **1**. We applied a freshly prepared solution of diazonium salt **1** in DMSO onto the graphene‐coated area of the glass slide and allowed it to react for 30 min in a closed chamber. The slide was then washed several times with methanol and dried under a gentle flow of nitrogen (Figure 2A).

To assess the extent of covalent functionalization, we performed Raman spectroscopy. We compared the Raman spectra of graphene before and after functionalization (Figure 2B). Unfunctionalized graphene exhibited a characteristic sharp and intense 2D peak representative of second‐order overtones of an in‐plane vibration, around ∼2683 cm^−1^, along with the G peak, corresponding to primary in‐plane vibrations at ∼1597 cm^−1^, indicating its sp^2^ planar and ordered structure. The D peak, associated with disorder, was generally absent due to lattice symmetries. Post‐functionalization, the defects in the graphene structure increased, resulting in the appearance of the D peak at ∼1344 cm^−1^, the D' peak at ∼1620 cm^−1^, and the D+G peak at ∼2947 cm^−1^. In Raman spectroscopy, the D‐band intensity (I_D_) is commonly correlated with the defect density and is evaluated relative to the G‐band intensity (I_G_). This comparison is valid because I_G_ is proportional to the amount of graphene carbon atoms illuminated within the laser spot and remains nearly constant for a single graphene layer up to a defect threshold corresponding to a mean inter‐defect distance of approximately 3 nm [43]. Given the large‐area and continuous monolayer nature of the graphene used in this study, and the mild reaction conditions employed (diazonium salt in DMSO at room temperature without physical agitation), we expect the formation of edge defects or vacancies to be minimal. We therefore attribute the observed increase in I_D_/I_G_ primarily to defects introduced by covalent modification of the graphene basal plane [44, 45, 46].

**FIGURE 3:**
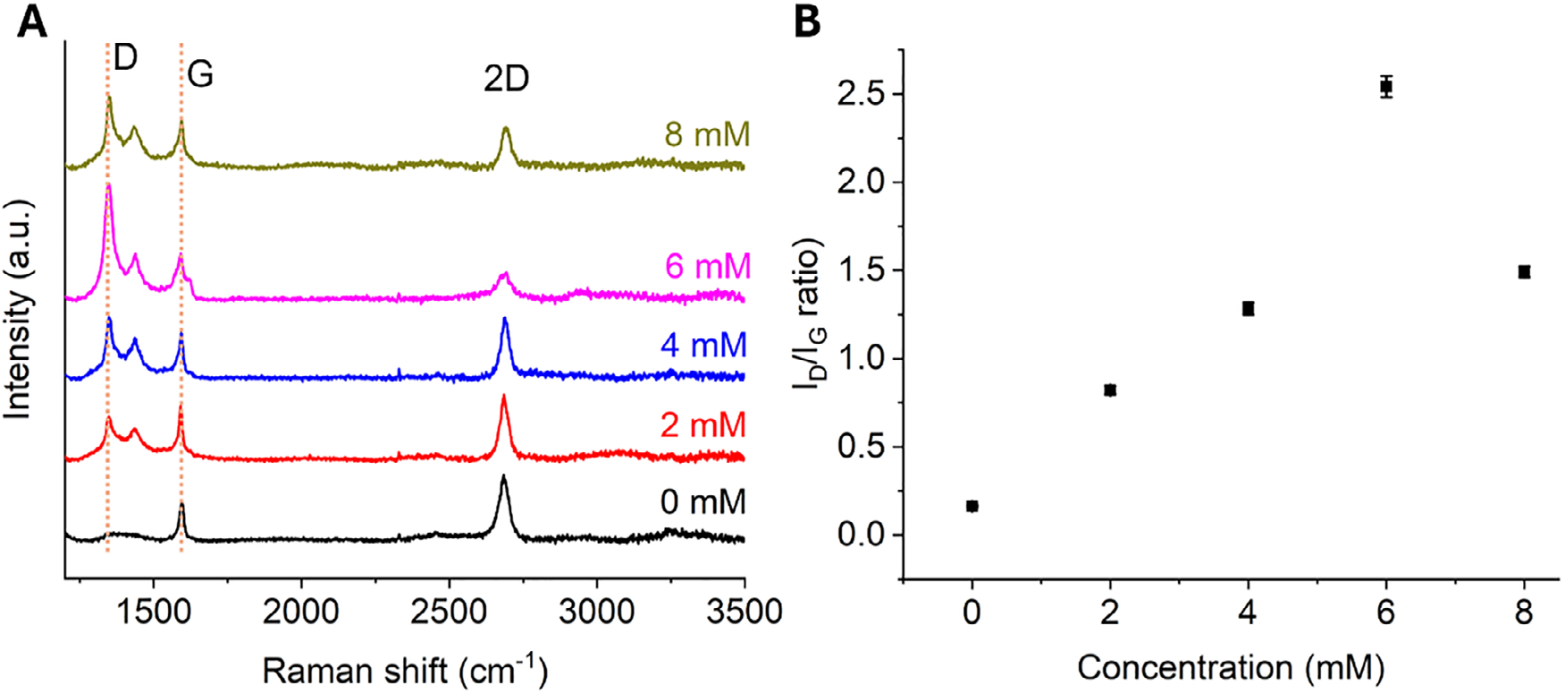
(A) Raman spectra of graphene on glass at different concentrations of diazonium salt **1**. (B) Graph illustrating the I_D_/I_G_ ratio in relation to concentration of the diazonium salt, with each data point representing the average of three independent measurements (error bars included).

To determine the optimal concentrations of diazonium salt **1** for maximizing the functionalization of graphene, we prepared four different concentrations (2, 4, 6, and 8 mm) of salt **1** in DMSO and evaluated their functionalization effectiveness. We compared the representative Raman spectra for each concentration with that of unfunctionalized graphene and calculated the *I_D_/I_G_* ratio. The degree of functionalization increased with the diazonium salt concentration up to 6 mm, at which the highest level (*I_D_/I_G_* = 2.54±0.06) was achieved. However, further increase in the salt concentration to 8 mm resulted in a reduction in the degree of functionalization (Figure 3). These findings suggest that an optimal concentration is critical for achieving the highest degree of functionalization. Beyond this threshold, steric hindrance and an increased propensity for side reactions involving diazonium salts may hinder effective functionalization, consistent with previous reports on carbon nanotube modification using diazonium chemistry [47]. The observed highest functionalization arises from a balance between site availability and the onset of deleterious side reactions.

To examine the homogeneity of the functionalization, we conducted Raman mapping over an area of approximately 140 µm x 90 µm and calculated the *I_D_/I_G_* ratio by measuring the intensities of the D and G bands for both unfunctionalized and functionalized graphene (Figure 2C–E). The Raman map clearly demonstrated the uniformity of functionalization: most spots on functionalized graphene displayed in light gray to white, whereas unfunctionalized graphene is shown in black. The average *I_D_/I_G_* ratio for unfunctionalized graphene was 0.07±0.03, while for TZ‐graphene it was 2.69±0.37. Based on the *I_D_/I_G_* ratio, we also calculated the defect distance (*L*_D_) and defect density (*n*_D_) according to the equations reported by Cançado et al. [48]. The *L*_D_ for Tz‐functionalized graphene was 7.3±1.15 nm, and the defect density (*n*_D_) was (6.05±1.89) x 10^11^ cm^−2^.

The successful functionalization of graphene with dipyridyl tetrazine moieties was further confirmed by x‐ray photoelectron spectroscopy (XPS) and UV–vis. The N 1s and S 2p peaks, which correspond to the nitrogen and sulfur atoms from dipyridyl tetrazine was evident in XPS of the functionalized graphene (Figure 2F,G). Due to the background absorption of glass, we examine the functionalization of graphene by UV–vis on quartz. Commercially available graphene on a quartz plate was used for this study. Following our standard protocol, the graphene‐coated quartz plate was immersed in 0.5 mL of a 6 mm diazonium salt solution of **1** in DMSO for 30 min, and then thoroughly rinsed with methanol to remove residual diazonium salt. The quartz plate was dried under a mild nitrogen flow. The functionalized graphene on quartz was then analyzed using UV–vis, and the results were compared to those of unfunctionalized graphene on quartz. The unfunctionalized graphene exhibited absorption around 268 nm, while the functionalized graphene displayed absorptions from both graphene and dipyridyl tetrazine (compound **5**, Figure S5), resulting in a strong peak at 268 nm with a broad shoulder from 350 to 600 nm (Figure 2H). Taken together, the spectroscopic data are consistent with functionalization of the graphene surface with the target tetrazine molecules.

### 2.4 The Tetrazine and TCO Ligation Proceeds on a Graphene Surface

To confirm the tetrazine ligation proceeds on the surface, we treated tetrazine functionalized graphene on a silicon wafer with a solution of TCO linked 2,3,5,6‐tetrafluorophenol (TCO‐PEG4‐TFP ester, 8 mm) in DMSO at room temperature followed by a methanol rinse. The silicon wafer was dried with a gentle stream of nitrogen followed by XPS analysis. The appearance of F 1s in addition to N 1s and S 2p was evident in the XPS indicating ligation took place on the tetrazine graphene surface (Figure S4).

### 2.5 Visualization of Target Molecule Capture by TIRF: Design and Evaluation of a Model Capture System

To assess the capture properties of the functionalized graphene, we designed single molecule pull down (SimPull) experiments [49] where we visualized the captured molecules via total internal reflection fluorescence (TIRF) microscopy [50, 51, 52], utilizing EGFP apo‐Ftn [53, 54, 55] as the model target molecule. Apo‐Ftn is a classic test molecule for cryo‐EM analysis due to its relatively large size, spherical shape, and high symmetry [56, 57, 58] and, by decoration with EGFP molecules, is a bright fluorescence reporter. Canonical mammalian apo‐Ftn consists of 24 subunits composed of 12 copies each of “heavy” and “light” chains [59].

**FIGURE 4:**
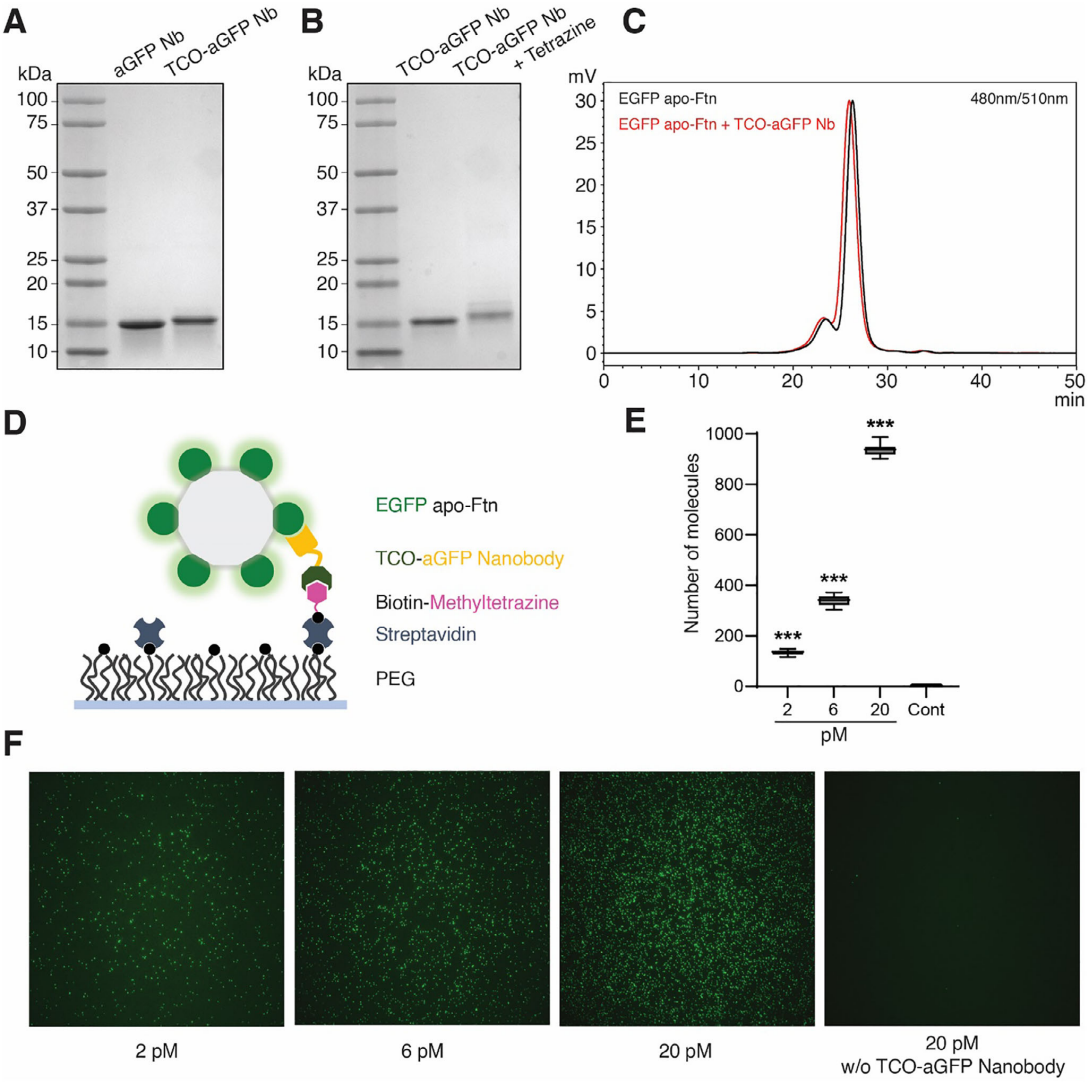
Chemical modification and SiMPull experiments on PEGylated TIRF slide. (A) SDS‐PAGE analysis of GFP nanobody before and after reaction with TCO‐PEG9‐maleimide labeling via a cysteine residue on the *N*‐terminus of the nanobody. The gel was visualized by Coomassie blue staining. (B) SDS‐PAGE analysis of TCO‐aGFP nanobody before and after incubation with methyltetrazine‐PEG4‐maleimide. (C) FSEC traces showing purified EGFP apo‐Ftn alone (black) and after incubation with TCO‐aGFP nanobody (red). EGFP fluorescence was monitored using excitation at 480 nm and emission at 510 nm. (D) Schematic representation of the capture of EGFP apo‐Ftn using TCO‐aGFP nanobody on a conventional TIRF slide, coated with tetrazine derivative. (E) SiMPull experiments of EGFP apo‐Ftn at varying concentrations. Control experiments to estimate nonspecific binding were performed without loading the TCO‐aGFP nanobody. (*n* = 15 images were analyzed; ^***^ *p*<0.001). (F) Representative TIRF micrographs from conditions shown in Figure 4E.

In the apo‐Ftn construct employed here, we concatenated the two chains into a single chain, added a *N*‐terminal EGFP tag, and expressed the resulting fusion construct in mammalian cells. To capture the EGFP apo‐Ftn on the tetrazine‐functionalized graphene, we employed an anti‐GFP nanobody [60], using its C‐terminal free cysteine for a maleimide reaction with a TCO‐tagged PEG compound, deemed TCO‐PEG9‐maleimide (MW 760 Da). Following derivatization of the nanobody by the TCO‐PEG‐maleimide moiety, we observe a small shift in the mobility of the protein on a SDS‐PAGE gel (Figure 4A). We also note that the TCO‐labeled anti‐GFP (TCO‐aGFP) nanobody reacts with the tetrazine compound, tetrazine‐PEG4‐maleimide (MW of 515), giving rise to a further retention of mobility on the gel (Figure 4B). Furthermore, fluorescence‐detection, size‐exclusion chromatography (FSEC) [61] demonstrates that the EGFP apo‐Ftn binds to the TCO‐aGFP nanobody (Figure 4C).

**FIGURE 5:**
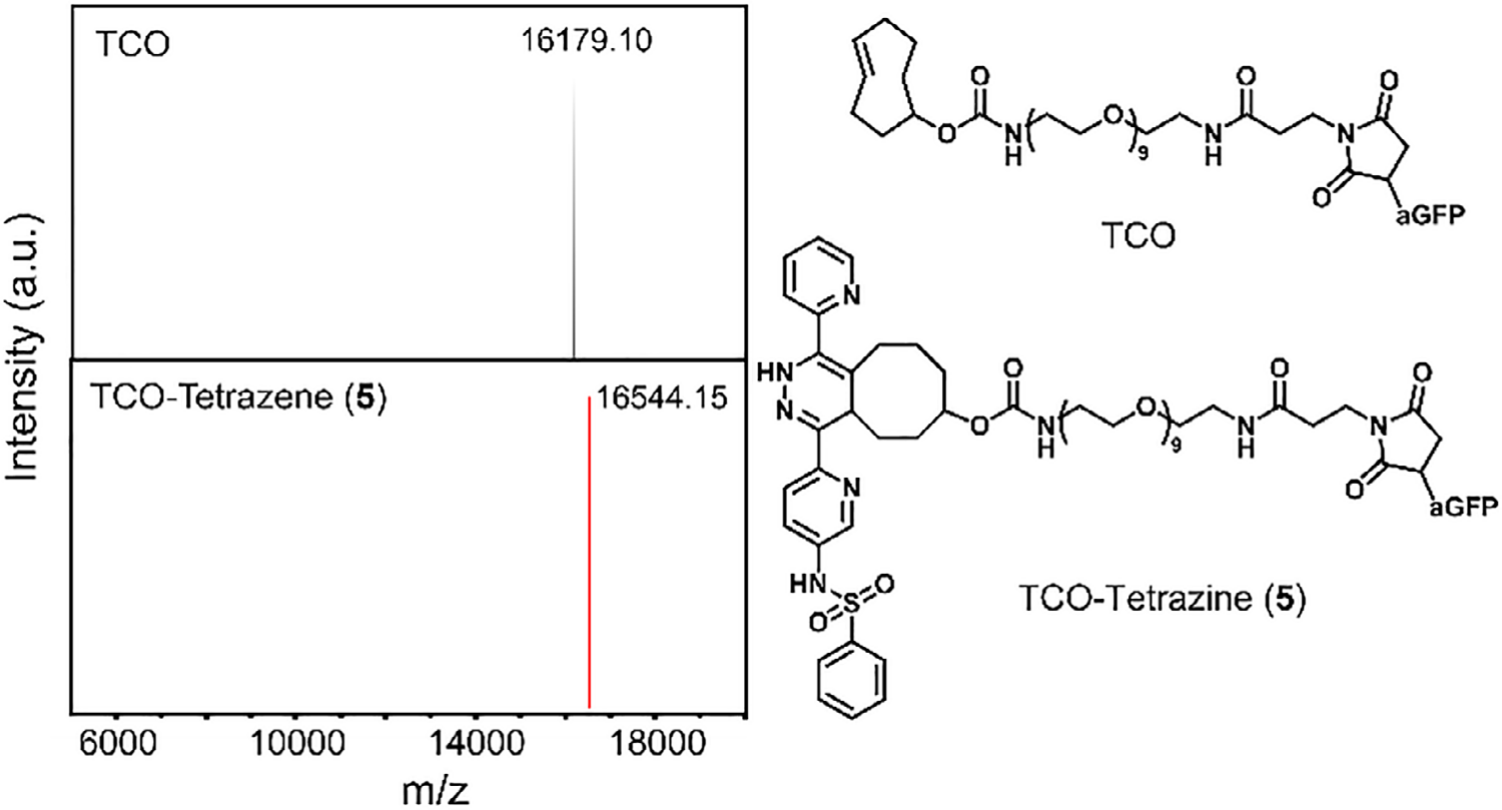
Deconvoluted ESI‐MS of TCO‐aGFP nanobody (black) and TCO‐aGFP‐tetrazine **5** conjugate (red) measured after 5 min of mixing at room temperature.

Prior to capturing biomacromolecules on the tetrazine‐modified surface, we confirmed that our solution model tetrazine **5** reacts with the TCO‐aGFP nanobody. The tetrazine‐TCO ligation was monitored using mass spectrometry, ESI‐MS. In this experiment, we utilized 1 equivalent of TCO‐aGFP nanobody (see Methods for the aGFP nanobody sequence) and 10 equivalents of tetrazine **5** to ensure complete consumption of TCO‐aGFP nanobody in the reaction. The reaction was carried out with 0.6 mg/mL of TCO‐aGFP in PBS buffer at pH ∼7.4 and 0.167 mg/mL of tetrazine **5** in DMSO. Mixing 10 µL of each solution resulted in an immediate color change from red to yellow, indicating the spontaneous reaction between tetrazine and TCO. The resulting mixture was analyzed by ESI‐MS. The ESI‐MS spectrum clearly shows full conversion to the tetrazine‐TCO conjugate (Figure 5). Comparison of the ESI‐MS for TCO‐aGFP nanobody and the tetrazine‐TCO conjugate also confirms the absence of the unreacted TCO‐aGFP nanobody (Figure S6), indicating a complete conversion.

Before testing the functionalized graphene, we wanted to verify if the TCO‐aGFP nanobody could capture EGFP apo‐Ftn on a TIRF slide and if the fluorescence from captured EGFP apo‐Ftn could be detected through TIRF microscopy. To test this, we functionalized a glass TIRF slide with the aGFP nanobody by “click” chemistry [62], using standard methods (Figure 4D). Using this pull‐down method via TIRF microscopy, we showed that we can successfully detect EGFP apo‐Ftn at picomolar concentrations on the glass slide (Figure 4E,F).

### 2.6 Functionalized Graphene on a Glass Slide Captures EGFP Apo‐Ftn

**FIGURE 6:**
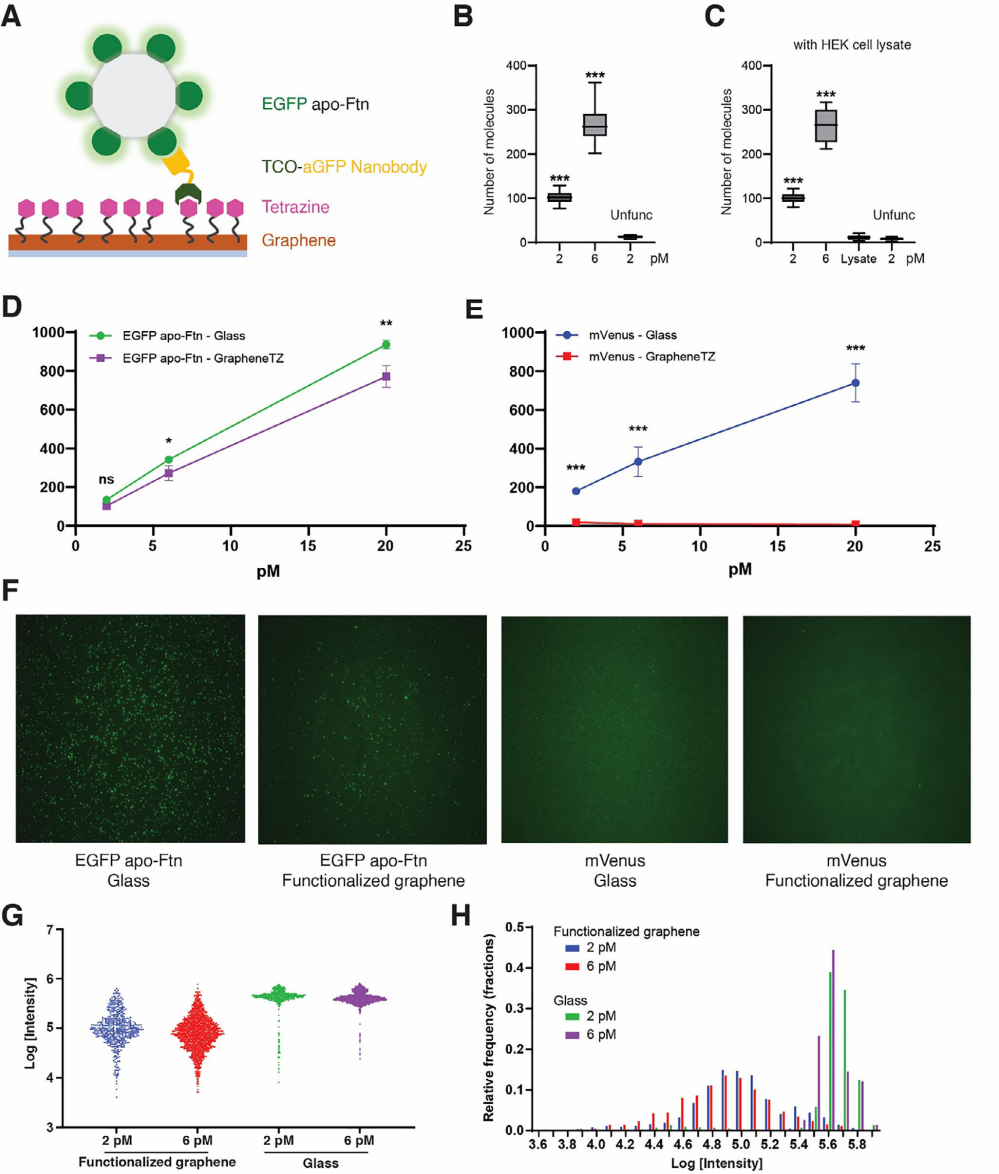
SiMPull experiments using a tetrazine‐functionalized graphene layer. (A) Schematic illustration of SiMPull experiments using tetrazine‐functionalized monolayer graphene, attached on a glass slide. (B) Quantification of the EGFP apo‐Ftn particles captured using the TCO‐aGFP nanobody covalently attached to the functionalized graphene layer. An unfunctionalized graphene layer was used as a control (*n* = 15 images analyzed). (C) Analysis of the capacity of the TCO‐aGFP nanobody functionalized graphene layer to capture EGFP apo‐Ftn in a crude mammalian cell lysate. Lysate only or unfunctionalized graphene were used as controls (*n* = 25 images analyzed). (D) Comparison of the ability of TCO‐aGFP nanobody to capture EGFP apo‐Ftn on either a typical TIRF slide or on a functionalized graphene monolayer. (E) Demonstration of the graphene quenching effect using monomeric mVenus protein captured on a conventional TIRF slide versus a functionalized graphene layer. (F) Representative TIRF micrographs from Figures 6D,E. EGFP apo‐Ftn or mVenus, at approximately 6 pm, were loaded onto either the conventional TIRF slide or the functionalized graphene. (G,H) Intensity distribution histograms of captured EGFP apo‐Ftn spots. The analysis was performed from 5 images from each condition.

To evaluate the ability of the tetrazine functionalized graphene to capture target molecules, we generated a small chamber similar to the standard SiMPull chamber using a monolayer of functionalized graphene attached to a cover slip with double‐sided tape and epoxy (Figure 6A). We successfully captured and visualized EGFP apo‐Ftn at picomolar concentrations on the functionalized graphene using the TCO‐aGFP nanobody (Figure 6B). Furthermore, we were able to capture the EGFP apo‐Ftn from a cell lysate, solubilized with detergent, which suggests the possible use of the functionalized graphene in the capture of target molecules from crude cell lysates or from other heterogeneous mixtures (Figure 6C).

To estimate the ‘background’ or nonspecific binding of the target molecule, we applied the EGFP apo‐Ftn to unfunctionalized graphene that had previously been incubated with the TCO‐aGFP nanobody. Imaging the resulting areas by TIRF demonstrated only a small extent of binding of EGFP apo‐Ftn to the unfunctionalized surface. Interestingly, the EGFP apo‐Ftn binding capacity of the functionalized graphene is slightly lower than the streptavidin‐coated capture surface of a canonical SiMPull chamber, based on comparison of the extents of binding at various concentrations (Figure 6D). We further noted that when we attempt to visualize the capture of a single mVenus protein on the functionalized graphene, we cannot detect the mVenus fluorescence signal, indicating the mVenus molecule is bound close to the graphene surface, and thus its fluorescence is quenched (Figure 6E,F) [63]. By contrast, the EGFP apo‐Ftn fluorescence is only partially quenched by the graphene because apo‐Ftn is approximately 140 Å in diameter and some of the EGFP molecules are far enough from the graphene surface to evade being quenched. This effect can be also seen in the EGFP apo‐Ftn signal from the graphene layer, which appears to have heterogeneous brightness and diminished signal compared to those from the glass slide (Figure 6G,H).

## 3 Conclusions

In summary, we have established a simple, mild, and efficient one‐step strategy for the covalent functionalization of monolayer CVD graphene with tetrazine moieties using diazonium chemistry. Covalent modification was confirmed by the appearance of characteristic defect bands in Raman spectra, and further validated by UV–vis and XPS elemental analyses. Notably, this method allows the quantification of surface functionalization density, which is determined to be (6.05±1.89) x 10^11^ cm^−2^ based on the Raman *I_D_/I_G_* ratio. Compared to other graphene modification approaches, our method yields a chemically well‐defined and highly reactive surface suitable for further applications. The tetrazine‐functionalized graphene readily undergoes bioorthogonal ligation with TCO‐tagged molecules, allowing for the covalent immobilization of various molecules. This was demonstrated through the successful conjugation of a TCO‐linked small molecule TFP ester and a TCO‐aGFP nanobody. Importantly, the nanobody functionalized graphene enabled the specific capture of EGFP‐tagged apo‐ferritin as low as 2 pm, which was the lowest concentration tested, even in complex cell lysate. Together, these results highlight the platform's significant potential for the specific protein capture using engineered nanobodies, with broad applications in biosensing, diagnostics, and nanobiotechnology.

## 4 Experimental Section

### 4.1 Tetrazine Reaction Kinetics

Solutions of TCO‐OH and **5** were prepared separately in a 1:1 mixture of spectroscopic grade THF and water. Initially, a UV–vis measurement of **5** in the THF:water solution was taken and allowed to stabilize for 10 min. Solutions of TCO‐OH (50, 100, and 200 µm) and **5** (5, 10, and 20 µm) were then mixed in quartz cuvettes in equal volumes (1:1) to achieve final concentrations of 2.5, 5, and 10 µm for 5 and 25, 50, and 100 µm for TCO‐OH, respectively. Reactions were monitored at 25°C using UV–vis spectroscopy, with time course measurements taken every two seconds for a total of 600 s. Each reaction concentration was conducted in triplicate, and data fitting showed a high degree of accuracy with an R^2^ value of 0.99.

The reaction between TCO‐OH and **5** was tracked by observing the decrease in absorption of **5** at 318 nm. The pseudo‐first‐order rate constants (k_obs_) were obtained by fitting the data to an exponential decay model. An increase in k_obs_ was observed as the concentration of **5** increased. The half‐life (t_1/2_) of **5** was calculated using t_1/2_ = ln2/k_obs_. The t_1/2_ values for 5, 10, and 20 µm tetrazine **5** are 68.3, 38.1, and 20.2 s, respectively (see Supporting Information).

### 4.2 Graphene Transfer

Graphene on copper foil was spin coated (2500 rpm, 1 min) with poly(methyl methacrylate) (PMMA, ∼550K MW, 8 wt% in anisole) and then dried in a fume hood overnight. Graphene on the backside was removed with oxygen (23 sccm, 4 min) and argon plasma (23 sccm, 2 min). Graphene was floated on a 0.1 m ammonium persulfate solution to remove the copper foil and several baths of ultrapure water (18.2 MΩ cm) to remove copper etchant. The resulting graphene was transferred to glass slides that were treated with 10 min of UV‐ozone and allowed to dry at room temperature overnight. PMMA was removed by submerging the glass slides in acetone.

### 4.3 Concentration of Diazonium Salt Estimation

The active content of the diazonium salt (compound **1**) was quantified using ^1^H NMR by employing an equimolar comparison with an internal standard, 1‐bromo‐4‐iodobenzene, in CD_3_OD. A solution containing 2.25 mg (0.00790 mmol) of the standard and 7.04 mg of the diazonium salt was prepared and analyzed by ^1^H NMR. Integration of the characteristic two aromatic proton signals corresponding to 1‐bromo‐4‐iodobenzene and the averaged six aromatic protons of compound **1** enabled the determination of their relative molar concentrations. Based on the integrated peak areas, it was determined that approximately 38% of the diazonium salt sample consisted of the active diazonium species. This value was subsequently used to determine the effective concentration of diazonium salt in DMSO solutions for further experiments.

### 4.4 TCO Labeled GFP Nanobody

The anti‐GFP nanobody, which has a C‐terminal octahistidine tag and a free cysteine residue, was expressed in One Shot BL21‐AI E. coli cells (Invitrogen). The protein was purified using “TALON (Takara Bio)” metal ion affinity chromatography followed by size‐exclusion chromatography (SEC). The purified nanobody was stored in phosphate buffered saline (PBS) containing 10% glycerol at −80°C. For “click” chemistry reactions, the purified anti‐GFP nanobody was first reduced with 1 mm of tris (2‐carboxyethyl) phosphine (TCEP) for 30 min. The reduced nanobody (2 mg/ml) was then incubated overnight with TCO‐PEG9‐maleimide (BP‐23872, BroadPharm) at a 20‐fold molar excess. Excess unreacted TCO reagent was removed using a desalting column (7K, Thermo Scientific). The TCO‐labeled nanobody (TCO‐aGFP nanobody) was then concentrated to 60 um using an Amicon Ultra Centrifugal Filter (Millipore). The final TCO conjugated nanobody was flash frozen in liquid nitrogen and stored at −80°C. The amino acid sequence for aGFP nanobody is: MQVQLVESGGALVQPGGSLRLSCAASGFPVNRYSMRWYRQAPGKEREWVAGMSSAGDRSSYEDSVKGRFTISRDDARNTVYLQMNSLKPEDTAVYYCNVNVGFEYWGQGTQVTVSSGLEVLFQGPAAAAHHHHHHHHGSC.

### 4.5 Expression and Purification of EGFP Apo‐Ftn

Baculovirus‐mediated expression was performed following standard protocol with minor modifications. HEK293S GnTIˉ cells were grown in suspension and transduced using P2 BacMam virus (1:20, v/v) encoding an EGFP‐mouse apo‐Ftn construct in which the light chain was convently fused to the heavy chain, and incubated at 37°C. At 12 h post‐transduction, 10 mm sodium butyrate was added to the culture and the temperature was shifted to 30°C. Cells were harvested 96 h post‐transduction by centrifugation and disrupted by sonication in Tris buffer saline (TBS) buffer (20 mm Tris‐Cl pH 8.0, 150 mm NaCl) containing protease inhibitors (1 mm PMSF, 0.05 mg/ml aprotinin, 2 µg/ml pepstatin A, and 2 µg/ml leupeptin). The supernatant was isolated by centrifugation and incubated with TALON resin at 4°C for 1 h. The resin was washed with 5 column volumes of TBS containing 10 mm imidazole, followed by 10 column volumes of TBS containing 40 mm imidazole. The protein was eluted with 5 column volumes of TBS containing 250 mm imidazole. Fractions of EGFP apo‐Ftn were pooled, concentrated, and further purified by SEC using a Superose 6 Increase 10/300 GL column (Cytiva) in phosphate buffered saline.

### 4.6 FSEC Analysis

In a volume of 80 uL, 1 nm of EGFP apo‐Ftn was incubated with or without TCO‐aGFP nanobody at a 10 fold molar excess. The samples were analyzed by SEC using a Superose 6 Increase 10/300 GL column (Cytiva) pre‐equilibrated with a buffer containing 20 mm Tris, 150 mm NaCl, and 0.02% glycol‐diosgenin (GDN). EGFP fluorescence was monitored using excitation at 480 nm and emission at 510 nm.

### 4.7 SiMPull Experiments

Coverslips and quartz slides for SiMPull experiments were prepared as described in Jain et al. [36]. Briefly, the coverslips and quartz slides were cleaned, passivated, and coated with a solution consisting of 50 mM methoxy polyethylene glycol (mPEG) and 1.25 mm biotinylated PEG in fresh sodium bicarbonate solution. A flow chamber was created by drilling 0.75 mm holes in a quartz slide using a diamond tip and placing double‐sided tape between the holes. A coverslip was placed on top of the slide and the edges were sealed with epoxy to create small flow chambers.

A solution of PBS containing 0.25 mg/mL streptavidin was applied to the slide, incubated at RT for 10 min, and washed off with PBS containing 0.25 mg/mL of bovine serum albumin (BSA). To capture TCO‐aGFP nanobody, 30 uL of 60 nm biotin‐PEG4‐methyltetrazine (BP‐22939, BroadPharm) in TBS buffer containing 0.02% GDN and 0.2 mg/mL BSA was applied to the slide, incubated for 10 min, and washed off with the wash buffer (TBS with 0.02% GDN and 0.2 mg/mL BSA). Subsequently, 30 uL of 200 nm TCO‐aGFP nanobody was applied to the slide, incubated at RT for 30 min, and then washed three times with the wash buffer. After applying 10 uL of each EGFP apo‐Ftn concentration to the chamber and incubating for 10 min, the slide was washed with 30 uL wash buffer and imaged using a Leica DMi8 TIRF microscope with an oil‐immersion 100× objective.

Regarding the verification of single capture, we relied on the well‐established biophysical properties of mVenus. The construct contains the A206K mutation, located on the surface of the mVenus protein, a substitution was widely known to prevent dimerization and enforce a monomeric state yet enable full retention of fluorescent properties [64]. Furthermore, the dimerization Kd of standard GFP variants is typically in the range of 100 um. In our experiments, the concentration of mVenus used for capture was in the picomolar (pM) range (∼107 times lower than the Kd). Under these dilute conditions, the formation of dimers or oligomers is thermodynamically implausible. Therefore, the individual fluorescent spots observed on the graphene surface can be confidently attributed to single mVenus molecules.

For SiMPull experiments on the graphene monolayer, the drilled quartz slide was extensively cleaned by sonication and methanol before chamber assembly. A graphene layer attached to the surface of a coverslip was placed on top of the drilled quartz slide using double‐sided tape, and the edges were sealed with epoxy. Rather than the streptavidin and methyltetrazine steps, 30 uL of 200 nm TCO‐aGFP nanobody was directly applied to the slide, incubated for 30 min, and then washed three times with the wash buffer. The EGFP apo‐Ftn application and imaging steps were performed as described above. To analyze the capture of EGFP apo‐Ftn from crude mammalian cell lysate, tsA201 cells were solubilized in TBS buffer containing 2% GDN and protease inhibitors (0.8 um aprotinin, 2 ug/mL leupeptin, and 2 um pepstatin) for 1 h at 4°C. The lysate was centrifuged for 1 h at 40 000 rpm to pellet insoluble material. The supernatant was then filtered through a 0.22 um syringe filter and added to each sample at a final cell concentration equivalent to 4 × 10^6^ cells/mL.

Images were captured using a back‐illuminated EMCCD camera (Andor iXon Ultra 888) with a 133 × 133 µm imaging area and a 13 µm pixel size. This 13 µm pixel size corresponds to 130 nm on the sample due to the 100× objective. Molecule quantitation and integrated intensity of the spots were determined using ComDet v0.5.5 in FIJI [51].

## Author Contributions

E.G. and M.H. designed the project. R.K.G. performed chemical synthesis and characterization, reaction kinetics, and graphene functionalization and characterization. R.K.G. and M.H. performed graphene transfer. M.H. performed XPS measurements. H.J. performed protein purification, modification of the aGFP nanobody, and the SiMPull experiments. A.G. expressed EGFP apo‐Ftn. All authors contributed to writing and editing the manuscript. All authors have given approval to the final version of the manuscript. M.H. acknowledges funding from the Arkansas Bioscience Institute.

## Conflicts of Interest

The authors declare no conflicts of interest.

## Supporting information




**Supporting File**: smll73236‐sup‐0001‐SuppMat.docx.

## Data Availability

The data that support the findings of this study are available from the corresponding author upon reasonable request.
