# Supplementary material for: Tetrazine Functionalized Graphene Enables Capture of Ultra‐Low Concentrations of Biomacromolecules
Source: Small. 2026 Mar 25;22(28):e13723. doi: 10.1002/smll.202513723 (PMC13181512; doi:10.1002/smll.202513723)
Supplement: Supplementary file 1 — Supporting File: smll73236‐sup‐0001‐SuppMat.docx. [file SMLL-22-e13723-s001.docx]

**Supporting Information**

**Tetrazine functionalized graphene enables capture of ultra-low concentrations of biomacromolecules**

*Ravindra K. Gupta, Hanbin Jeong, April Goehring, Eric Gouaux, Maggie He**

R. K. Gupta, M. He

Department of Chemistry and Biochemistry, University of Arkansas, Fayetteville, AR, USA

E-mail: maggiehe@uark.edu

H. Jeong, A. Goehring, E. Gouaux

Vollum Institute, Oregon Health and Science University, Portland, OR, USA

A. Goehring, E. Gouaux

Howard Hughes Medical Institute, Oregon Health and Science University, Portland, OR, USA

# General methods

All starting materials and reagents were procured from commercial vendors (where noted) and used as obtained unless otherwise specified. 5-amino-2-cyanopridine, 2-cyanopyridine and 4-acetamidobenzenesulfonyl chloride were purchased from Ambeed, Inc. Hydrazine monohydrate, phenyliodonium diacetate, pyridine, benzenesulfonyl chloride and poly(methyl methacrylate) were purchased from Beantown Chemicals. Ammonium persulfate was purchased from Across chemicals. TCO-PEG4-TFP ester was purchased from Broadpharm. Dichloromethane (CH_2_Cl_2_), ethyl acetate (EtOAc), methanol and hexanes (Hex) were purchased from VWR. Graphene on Cu foil was purchased from Graphenea. Column chromatography was performed using SiliaFlash® P60 (particle size 40-63 μm, SILICYCLE Inc.). All of the compounds were characterized by ^1^H and ^13^C nuclear magnetic resonance (NMR), mass spectrometry and FTIR spectroscopy. NMR spectra were obtained on a Bruker Advance 400 MHz spectrometer. Chemical shifts (δ) in ^1^H NMR were reported in parts per million (ppm) in DMSO-d_6_ (2.50 ppm) at 298k. Chemical shifts (δ) in ^13^C NMR were reported in parts per million (ppm) signals in DMSO-d_6_ (39.52 ppm) and MeOD-d_4_ (49 ppm) at 298k. NMR splitting parameters are indicated as follows: s, singlet; d, doublet; m, multiplet; dd, doublet of doublet; td, triplet of doublet. HRMS mass spectra were acquired using Shimadzu IT-TOF. IR measurement was performed FTIR spectrometer (Agilent, Cary 630, Agilent USA). Raman spectroscopy was performed by RENISHAW inVia Raman microscope using a 532 nm laser. UV-Vis absorption in solution and on quartz plates were recorded on a JASCO V-770 spectrophotometer. XPS measurements were performed on a Physical Electronics Versaprobe II X-ray photoelectron spectrometer with a hemispherical energy analyzer and a monochromatic X-ray source (Al Kα, 1486.6 eV).

1. **Tetrazene-TCO ligation experiment**

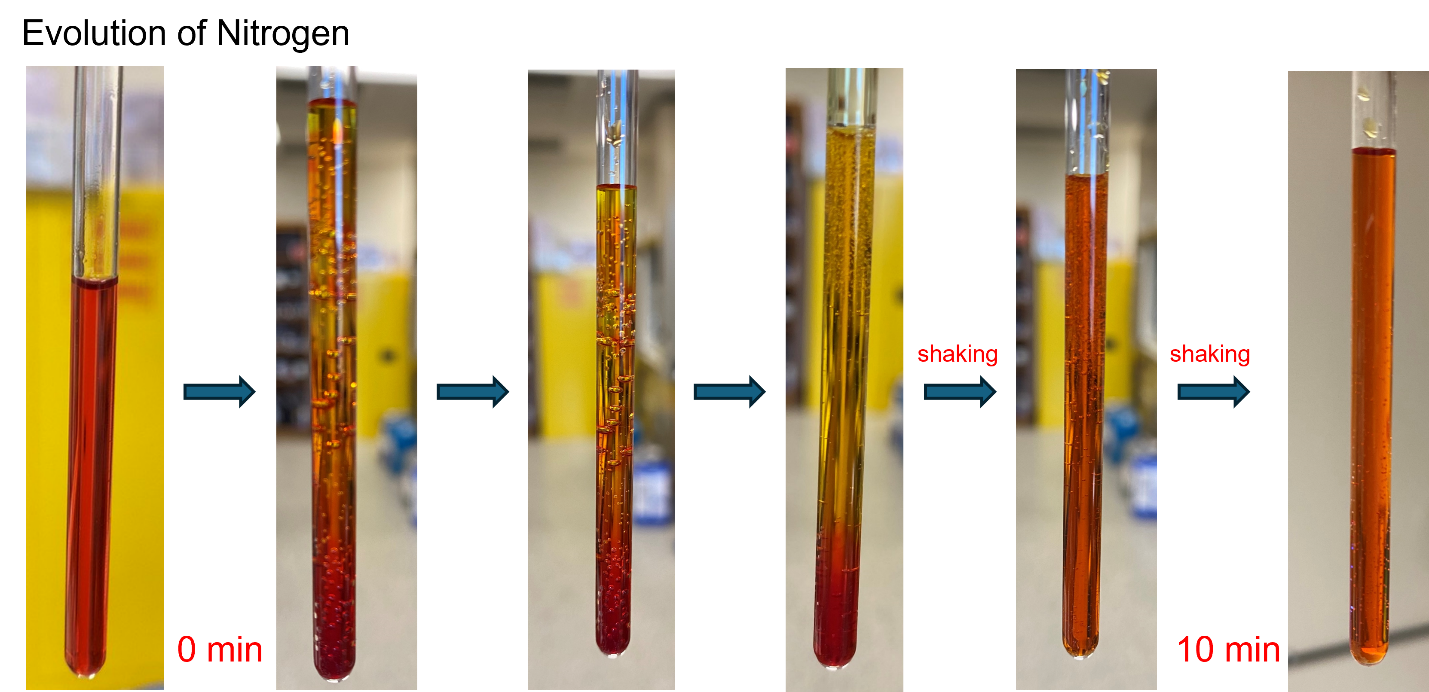


**Figure S1.** Evolution of Nitrogen gas bubble from ^1^H NMR experiment of TCO-OH and Compound **5** in (CD_3_)_2_SO, 400 MHz.


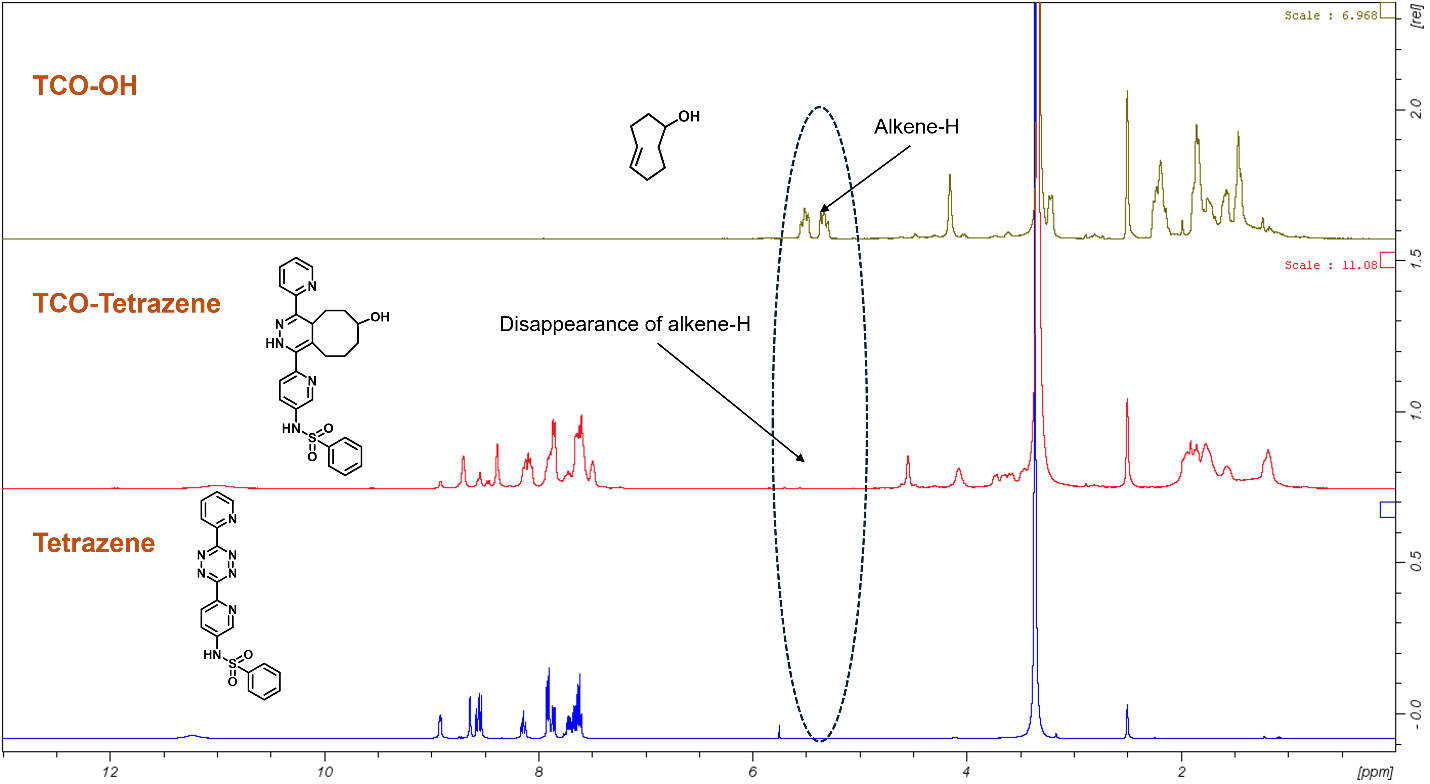


**Figure S2.** ^1^H NMR experiment for TCO-OH and tetrazene **5** reaction in (CD_3_)_2_SO, 400 MHz.

1. **Tetrazene-TCO reaction kinetics**

**
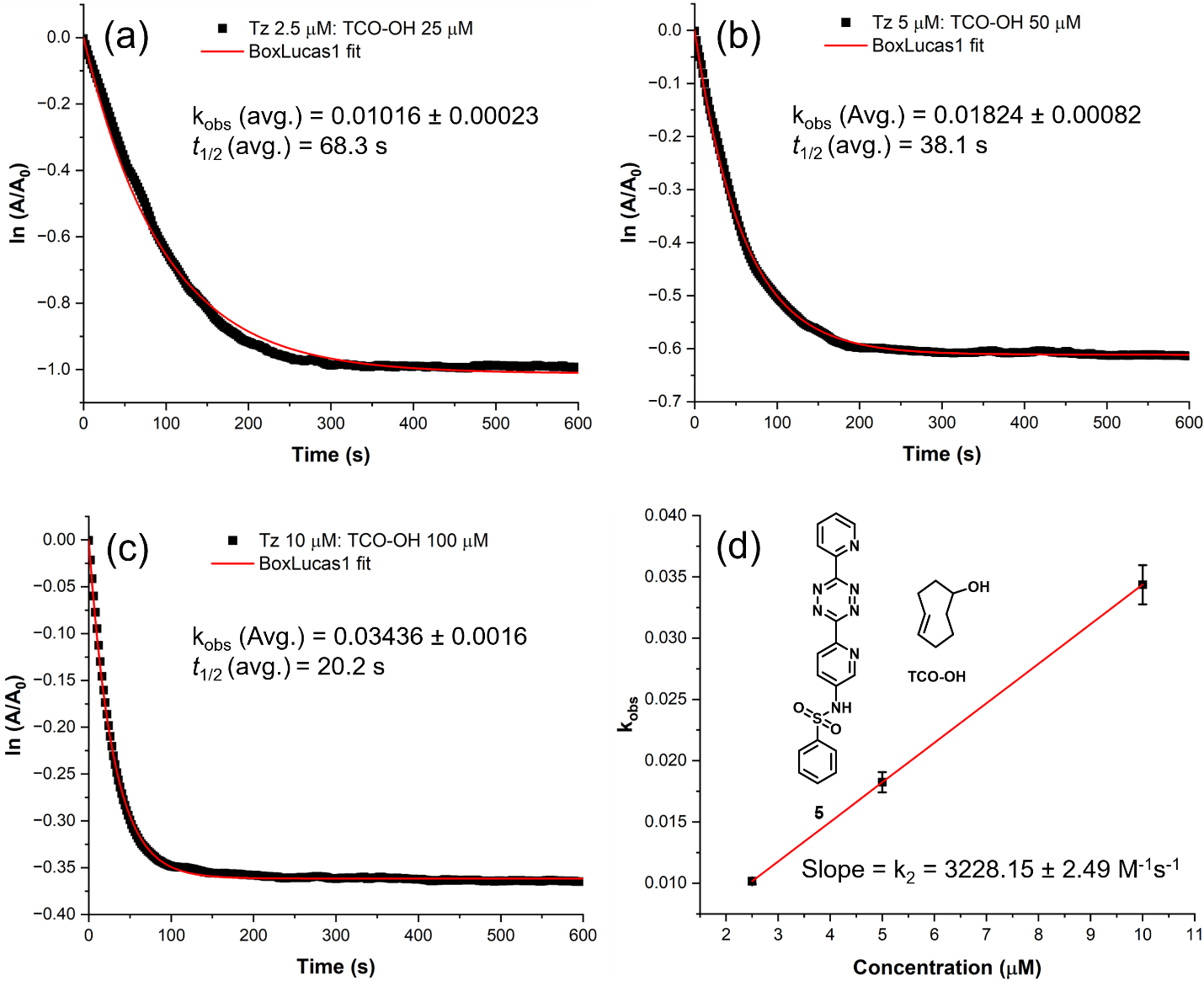
**

**Figure S3.** Representative spectra (a, b, and c) showing the decrease in absorbance at 318 nm for compound **5** over time at different concentrations, with an average k_obs_ and t_1/2_, in a 1:1 ratio of THF and water; a) 2.5 µM of **5** with 25 µM of TCO-OH; b) 5 µM of **5** with 50 µM of TCO-OH; c) 10 µM of **5** with 100 µM of TCO-OH; d) Plot of k_obs_ versus the concentration of **5**, with each data point representing the average of three independent runs (error bars included). The second-order rate constant (k_2_ = 3228.15 ± 2.49 M^-1^s^-1^) was derived from the slope of this graph.

1. **XPS of tetrazine ligation on graphene**

**Figure S4.** Survey scan of tetrazine ligation between TCO-PEG4-TFP ester and TZ-graphene.

1. **UV-Vis spectrum of 5**

**
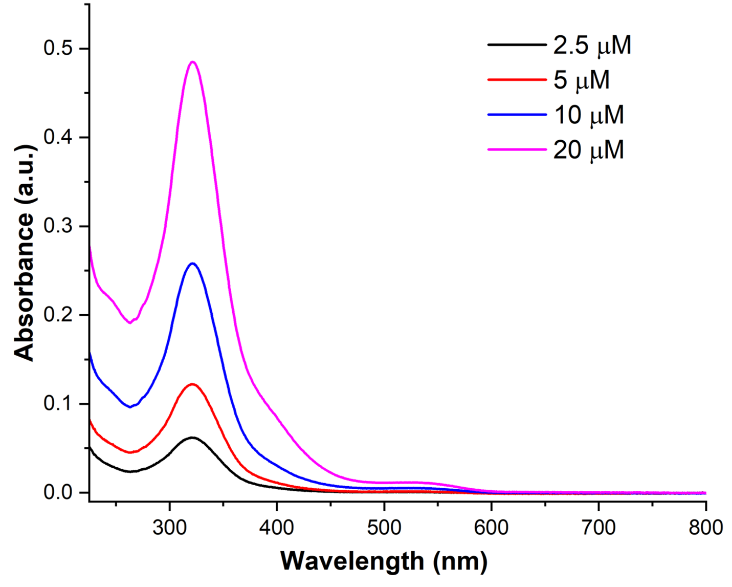
**

**Figure S5.** UV-Vis spectrum of compound **5** in THF:water (1:1) solution in various concentrations at room temperature.

1. **Monitoring the tetrazine ligation of tetrazine 5 and TCO-aGFP nanobody by ESI-MS**

**Figure S6.** ESI-MS of TCO-aGFP (red) and TCO-aGFP-tetrazine **5** conjugate (green).

1. **Synthetic procedures and compound characterization**

Synthesis of compound **3**^1^

5-Amino-2-cyanopyridine (3.40 g, 28.8 mmol) was added to 2-cyanopyridine (3.00 g, 28.8 mmol). Then hydrazine monohydrate (99%, 5.85 mL, 155 mmol) was added and the reaction mixture was refluxed under nitrogen atmosphere for 12 h. After cooling to room temperature, ice cold water (120 mL) was added. The formed solid was filtered on a Büchner funnel, then rinsed with ice-cold water (120 mL). The collected solid was transferred to a flask and suspended in methylene chloride (160 mL). Phenyliodonium diacetate (PIDA, 9.65 g, 30 mmol) was added to the stirring suspension and stirred at room temperature for 45 min. The reaction mixture was adsorbed onto silica gel (20 g) and the product was purified by column chromatography with polarity of the eluent gradually increased from dichloromethane, dichloromethane: ethyl acetate (10:20) to dichloromethane: methanol (10:1). Finally, the solid was washed with ethyl acetate to afford **3** as red solid (1.6 g, 22%), followed by **4** as magenta colored solid (0.82 g, 12%). Analytical data matched with reported literature.

**3:** ^1^H NMR (400 MHz, (CD_3_)_2_SO): δ 8.90-8.89 (m, 1H), 8.54-8.51 (m, 1H), 8.37-8.35 (m, 1H), 8.24-8.23 (m, 1H), 8.12 (td, *J*= 7.6, 2 Hz, 1H), 7.70-7.67 (m, 1H), 7.12 (dd, *J*= 8.8, 2.8 Hz, 1H), 6.37 (ArNH_2_, s, 2H); ^13^C NMR (100 MHz, (CD_3_)_2_SO): δ 162.9, 162.6, 150.5, 148, 137.7, 137.3, 136.1, 126.2, 125.7, 123.7, 119; FTIR (ATR, ʋ cm^-1^): 3377, 3325, 3196, 3092, 1653, 1580, 1431, 1392, 1290, 1124, 1047, 999, 919.

**4:** FTIR (ATR, ʋ cm^-1^): 3094, 3054, 1577, 1439, 1385, 1323, 1254, 1127, 1090, 991, 917.

Synthesis of compound **2**^2^

To an ice-cooled solution of **3** (0.5 g, 1.99 mmol) in 8 mL of pyridine was added dropwise 4-acetamidobenzenesulfonyl chloride (0.93 g, 3.98 mmol) in 2 mL pyridine. The mixture was stirred at 0 °C for 2 h and allowed to reach room temperature. Water (20 mL) was added and the solid was filtered and dried under vacuum. The crude product was purified by dissolving in minimum methanol and precipitated by adding dichloromethane to give a pink solid, compound **2** (0.75 g, 84%). Analytical data matched with reported literature.

**2:** ^1^H NMR (400 MHz, (CD_3_)_2_SO): δ 11.10 (s, NH, 1H), 10.35 (s, NH, 1H), 8.92-8.90 (m, 1H), 8.63-8.62 (m, 1H), 8.58-8.53 (m, 2H), 8.14 (td, *J*= 7.8, 1.6 Hz, 1H), 7.85-7.82 (m, 3H), 7.76-7.74 (m, 2H), 7.73-7.69 (m, 1H), 2.04 (s, 3H); ^13^C NMR (100 MHz, (CD_3_)_2_SO): δ 169.2, 163.1, 162.7, 150.6, 150.1, 144.7, 143.7, 141.1, 137.8, 137.3, 132.2, 128.2, 126.6, 126.3, 125.1, 124.2, 118.8, 24.1; FTIR (ATR, ʋ cm^-1^): 3254, 3181, 3105, 3053, 1694, 1584, 1532, 1394, 1370, 1318, 1252, 1159, 1090, 997, 906.

Synthesis of compound **1**^2^

Compound **2** (0.209 g, 0.47 mmol) was dissolved in 15 mL concentrated HCl and heated to 50 °C overnight. Then the solution was cooled to room temperature and concentrated HCl (10 mL) and water (20 mL) was added. The mixture was cooled to 0 °C and an aqueous solution of NaNO_2_ (0.26 g, 3.77 mmol) was added dropwise. After 1.5 h, reaction mixture was poured into cold acetone (400 mL). The precipitate was filtered and washed with cold acetone (50 mL), yielding compound **1,** an orange solid (0.175 g, 83%). Compound **1** was stored under vacuum at -23 °C to prevent decomposition.

**1:** ^1^H NMR (400 MHz, (CD_3_)_2_SO): δ 12.66 (s, NH, 1H), 8.97 (d, *J*= 9 Hz, 2H), 8.93-8.92 (m, 1H), 8.85 (d, *J*= 2.4 Hz, 1H), 8.60-8.55 (m, 2H), 8.52 (d, *J*= 9 Hz, 2H), 8.16 (td, *J*= 7.8, 2 Hz, 1H), 8.04 (dd, *J*= 8.7, 2.6 Hz, 1H), 7.75-7.72 (m, 1H); ^13^C NMR (100 MHz, CD_3_OD): δ 164, 160.9, 151.2, 149.1, 145.6, 145.1, 144.9, 142.2, 139.2, 135.2, 131.5, 130.8, 130.5, 127.8, 127.7, 121.9; HRMS (ESI-MS) m/z: [M]^+^ calculated for C_18_H_12_N_9_O_2_S^+^ 418.0829; found: 418.0837; FTIR (ATR, ʋ cm^-1^): 3466, 3284, 3092, 2300, 1612, 1556, 1452, 1398, 1370, 1308, 1167, 1073, 921.

Synthesis of compound **5**

To an ice-cooled solution of **3** (0.1 g, 0.4 mmol) in 3 mL pyridine was added dropwise benzenesulfonyl chloride (0.101 mL, 0.8 mmol). The mixture was stirred at 0 °C for 2 h and allowed to reach room temperature. Water (15 mL) was added, and the solid was filtered and dried under vacuum. The crude product was purified by dissolving in minimum methanol and precipitated by adding ether to give compound **5,** a pink solid (0.095 g, 61%).

**5:** ^1^H NMR (400 MHz, (CD_3_)_2_SO): δ 11.23 (s, NH, 1H), 8.93-8.92 (m, 1H), 8.64-8.63 (m, 1H), 8.58-8.53 (m, 2H), 8.14 (td, *J*= 7.6, 1.6 Hz, 1H), 7.92-7.90 (m, 2H), 7.86 (dd, *J*= 8.7, 2.7 Hz, 1H), 7.73-7.60 (m, 4H); ^13^C NMR (100 MHz, (CD_3_)_2_SO): δ 163, 162.6, 150.6, 150.1, 144.8, 141.2, 138.9, 137.7, 137.2, 133.5, 129.6, 126.7, 126.6, 126.4, 125,124.2; HRMS (ESI-MS) m/z: [M+H]^+^ calculated for C_18_H_14_N_7_O_2_S^+^ 392.0924; found: 392.0922; FTIR (ATR, ʋ cm^-1^): 3101, 3043, 1580, 1506, 1390, 1331, 1252, 1217, 1193, 1131, 1090, 913.

1. **NMR Spectra:**


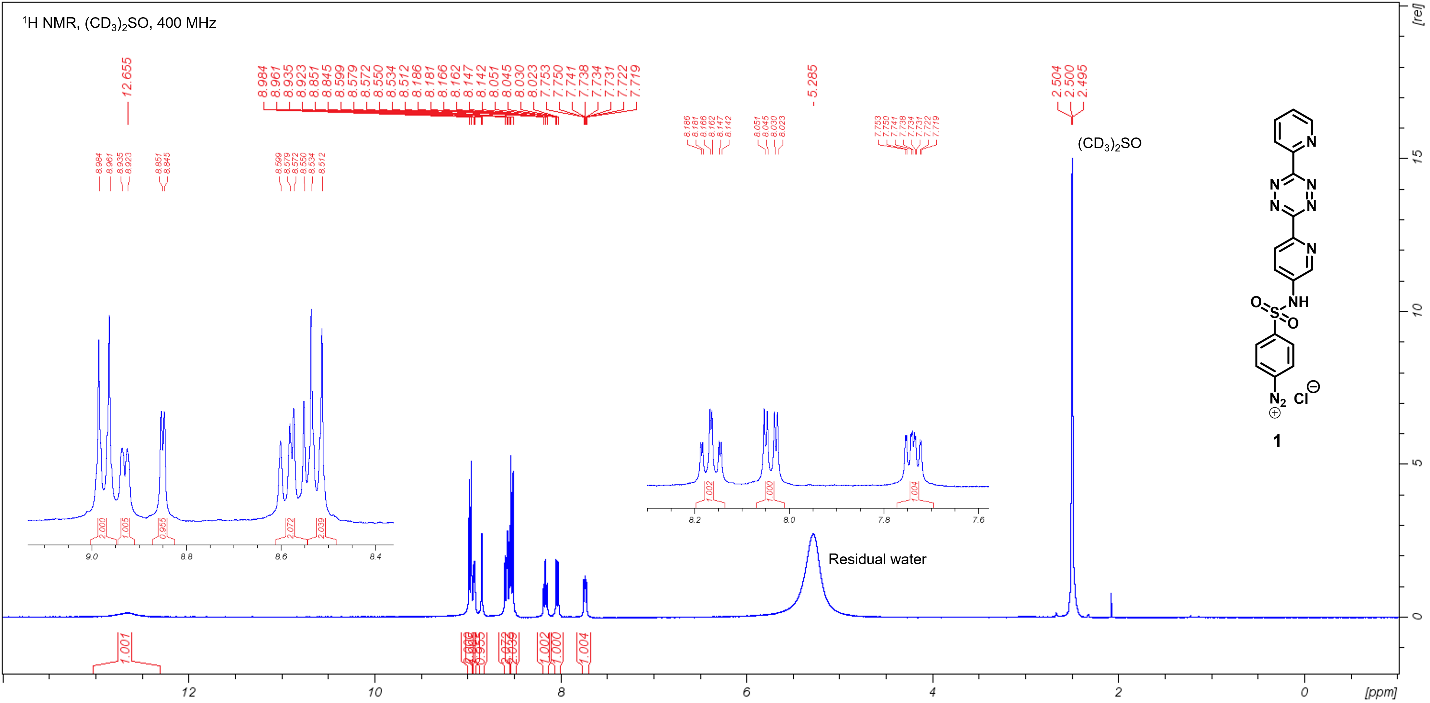


**Figure S7.** ^1^H NMR (400 MHz, (CD_3_)_2_SO) spectrum of **1**.


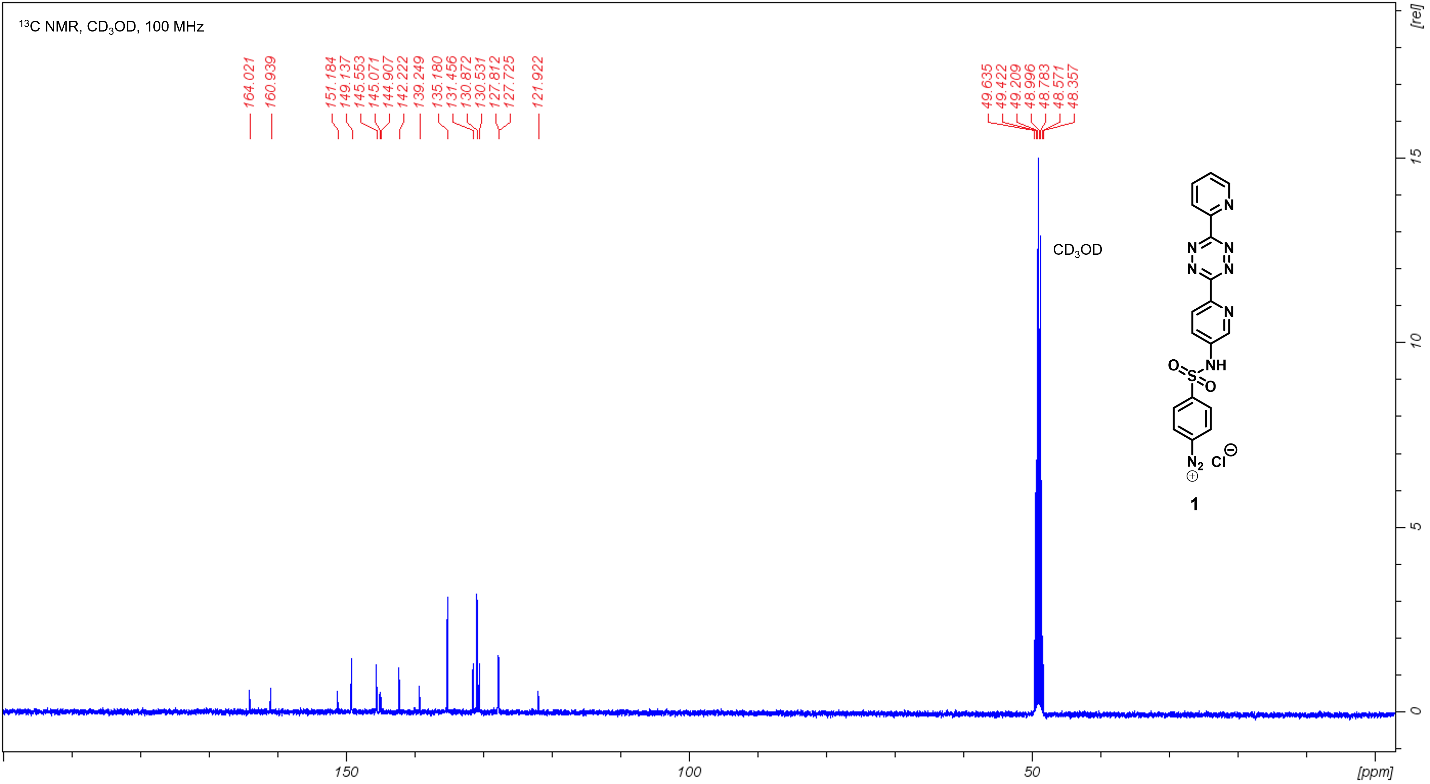
**Figure S8.** ^13^C NMR (100 MHz, CD_3_OD) spectrum of **1**.


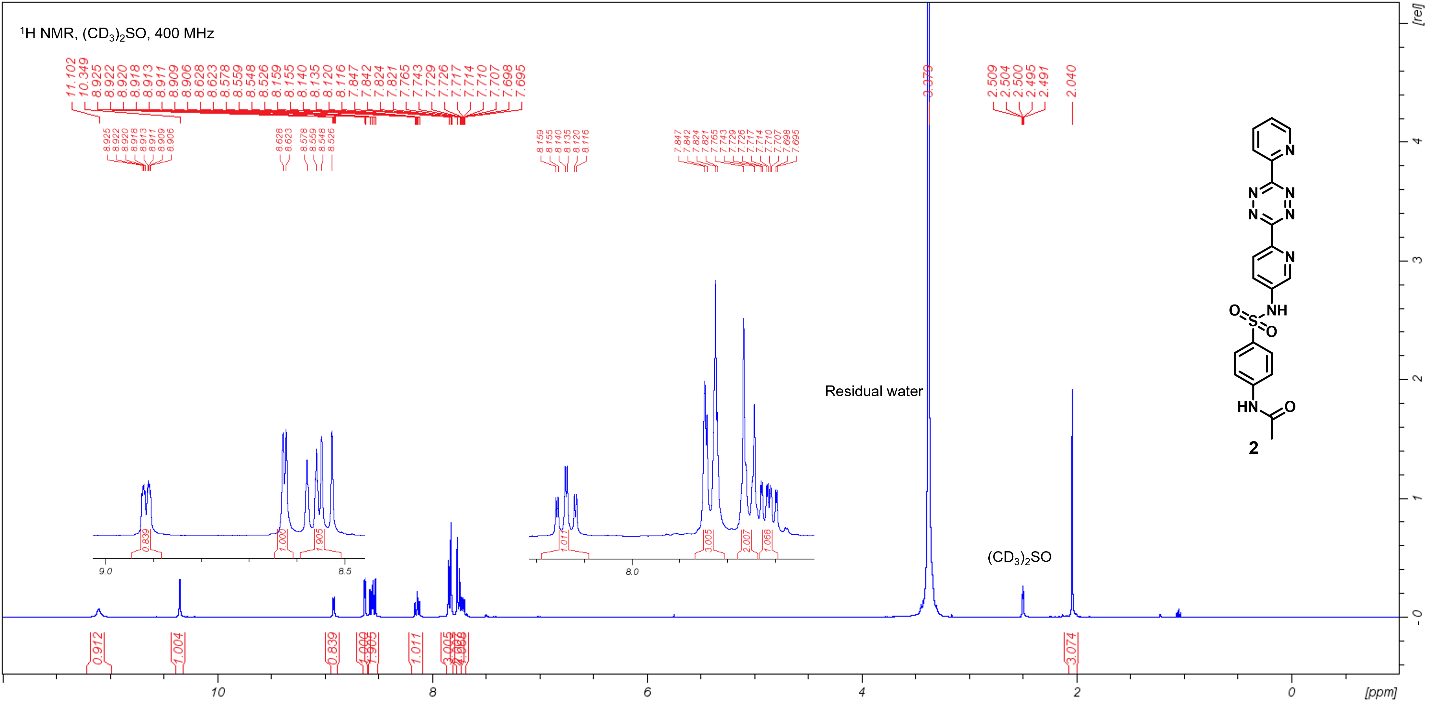


**Figure S9.** ^1^H NMR (400 MHz, (CD_3_)_2_SO) spectrum of **2**.


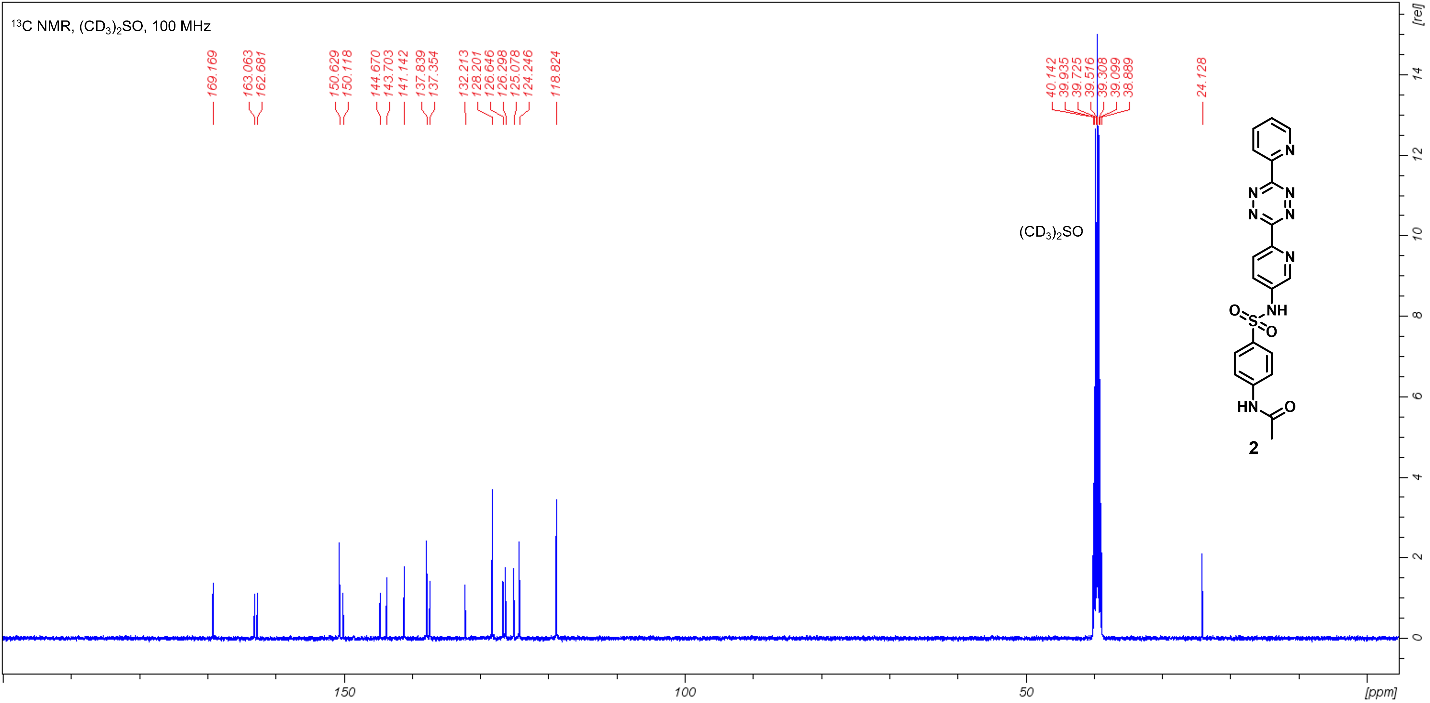


**Figure S10.** ^13^C NMR (100 MHz, (CD_3_)_2_SO) spectrum of **2**.


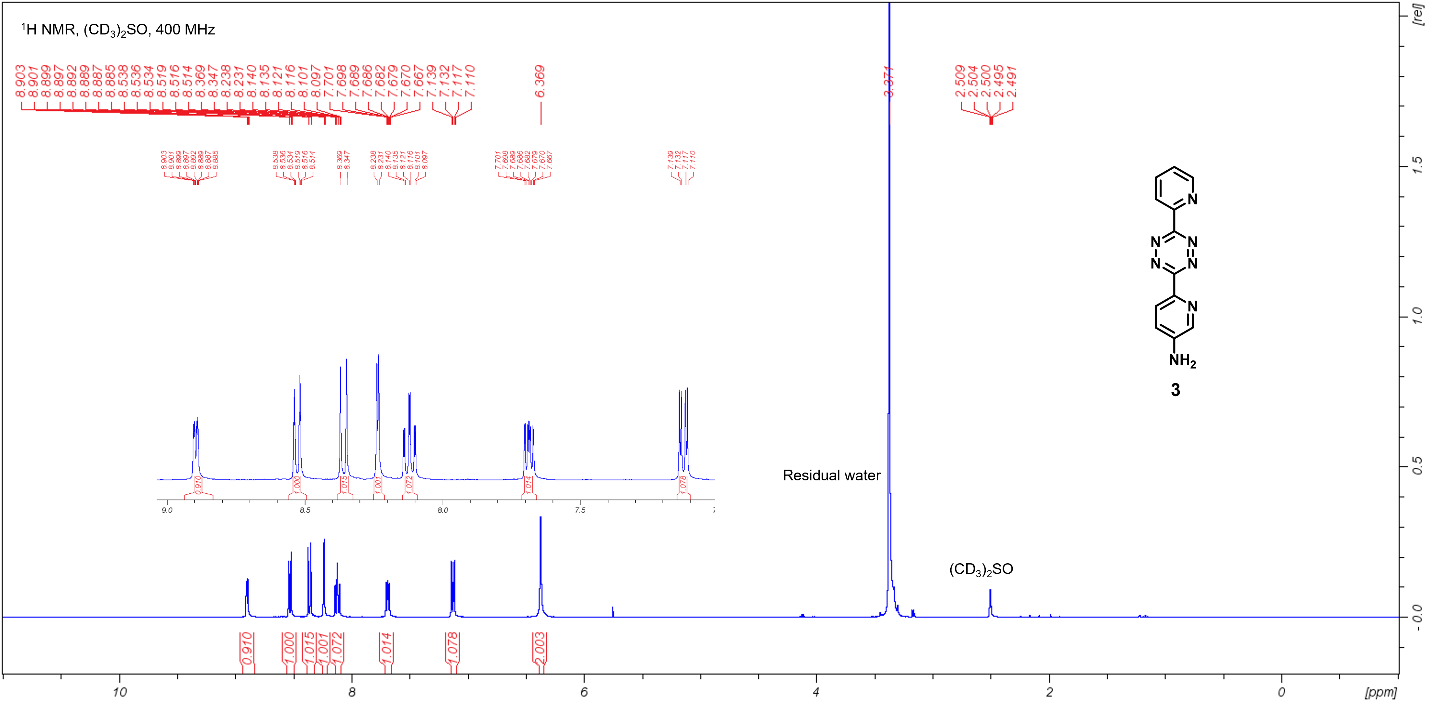


**Figure S11.** ^1^H NMR (400 MHz, (CD_3_)_2_SO) spectrum of **3**.


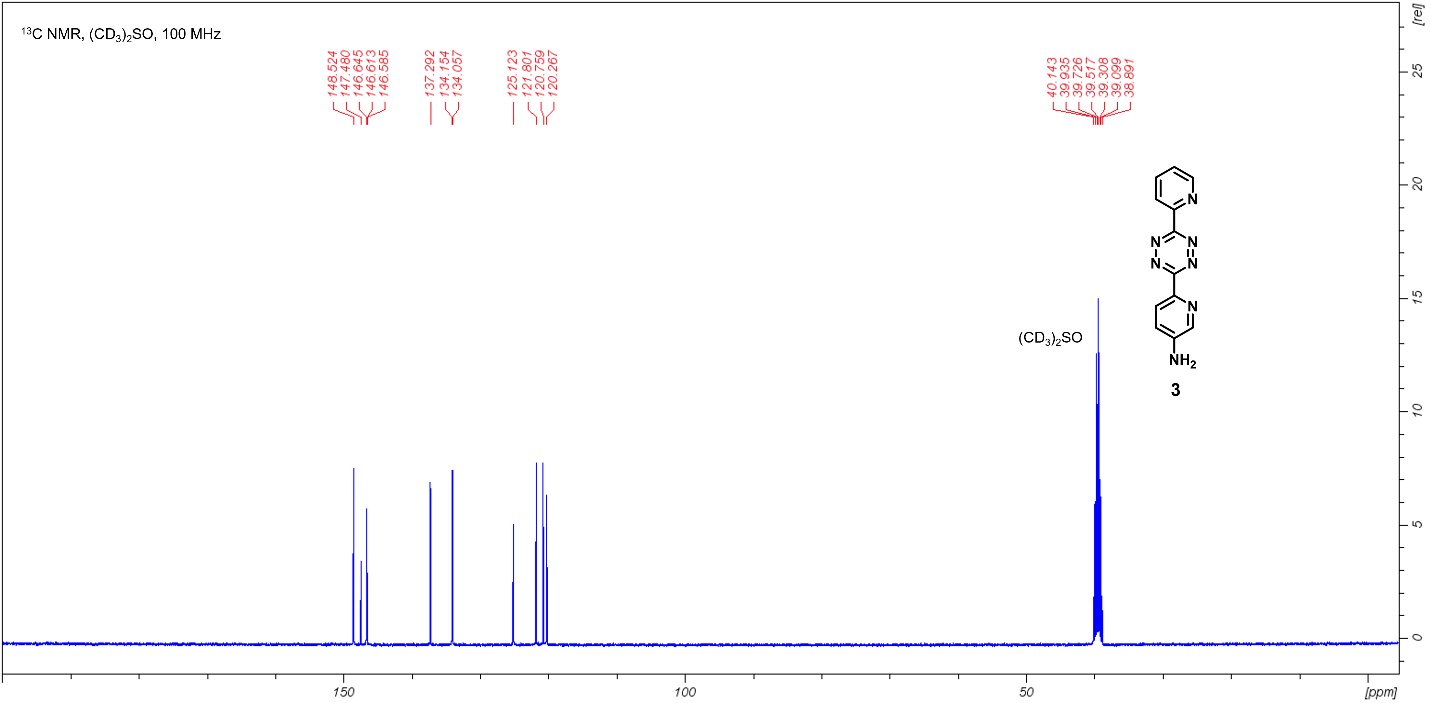


**Figure S12.** ^13^C NMR (100 MHz, (CD_3_)_2_SO) spectrum of **3**.

**
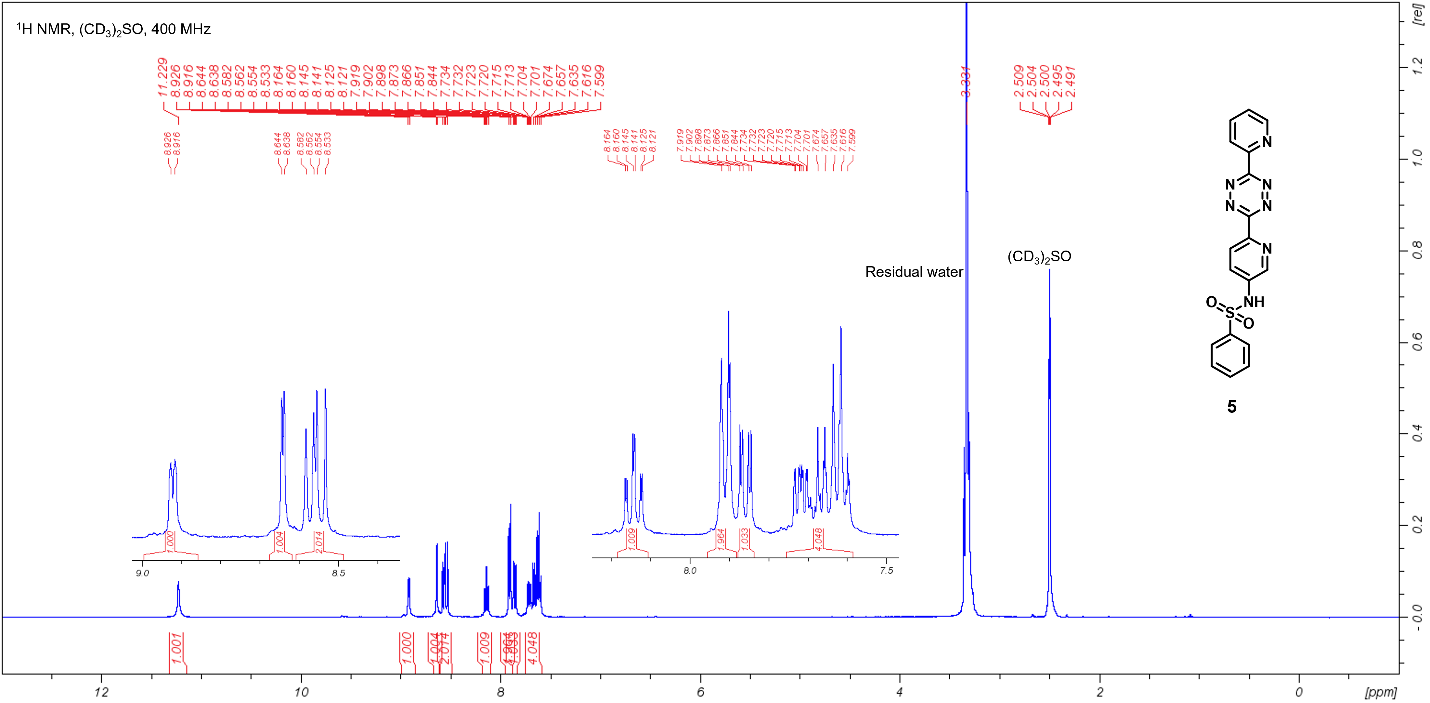
**

**Figure S13.** ^1^H NMR (400 MHz, (CD_3_)_2_SO) spectrum of **5**.

**
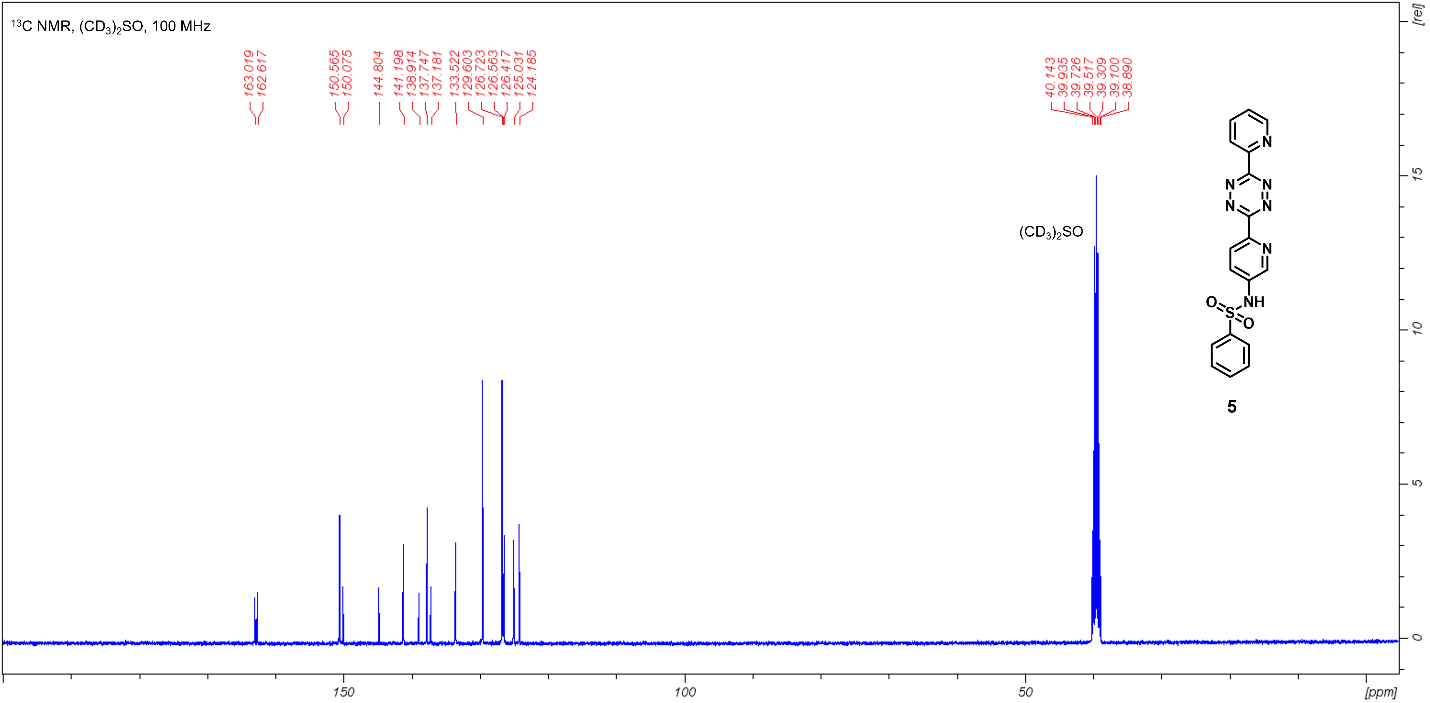
**

**Figure S14.** ^13^C NMR (100 MHz, (CD_3_)_2_SO) spectrum of **5**.

**References:**

(1) Briš, A.; Murata, Y.; Hashikawa, Y.; Margetić, D. Utilization of sym-tetrazines as guanidine delivery cycloaddition reagents. An experimental and computational study. *J. Mol. Struct.* **2023**, *1272*, 134207.

(2) Zhang, J.; Men, Y.; Lv, S.; Yi, L.; Chen, J.-F. Protein tetrazinylation via diazonium coupling for covalent and catalyst-free bioconjugation. *Org. Biomol. Chem.* **2015**, *13* (47), 11422-11425.
